# Extended string-like binding of the phosphorylated HP1α N-terminal tail to the lysine 9-methylated histone H3 tail

**DOI:** 10.1038/srep22527

**Published:** 2016-03-03

**Authors:** Hideaki Shimojo, Ayumi Kawaguchi, Takashi Oda, Nobuto Hashiguchi, Satoshi Omori, Kei Moritsugu, Akinori Kidera, Kyoko Hiragami-Hamada, Jun-ichi Nakayama, Mamoru Sato, Yoshifumi Nishimura

**Affiliations:** 1 Graduate School of Medical Life Science, Yokohama City University, 1-7-29 Suehiro-cho, Tsurumi-ku, Yokohama, Kanagawa 230-0045, Japan; 2 Division of Genome Technologies, RIKEN Center for Life Science Technologies, 1-7-22 Suehiro-cho, Tsurumi-ku, Yokohama, Kanagawa 230-0045, Japan; 3 Graduate School of Natural Sciences, Nagoya City University, 1 Yamanohata, Mizuho, Nagoya, Aichi 467-8501, Japan

## Abstract

The chromodomain of HP1α binds directly to lysine 9-methylated histone H3 (H3K9me). This interaction is enhanced by phosphorylation of serine residues in the N-terminal tail of HP1α by unknown mechanism. Here we show that phosphorylation modulates flexibility of HP1α’s N-terminal tail, which strengthens the interaction with H3. NMR analysis of HP1α’s chromodomain with N-terminal tail reveals that phosphorylation does not change the overall tertiary structure, but apparently reduces the tail dynamics. Small angle X-ray scattering confirms that phosphorylation contributes to extending HP1α’s N-terminal tail. Systematic analysis using deletion mutants and replica exchange molecular dynamics simulations indicate that the phosphorylated serines and following acidic segment behave like an extended string and dynamically bind to H3 basic residues; without phosphorylation, the most N-terminal basic segment of HP1α inhibits interaction of the acidic segment with H3. Thus, the dynamic string-like behavior of HP1α’s N-terminal tail underlies the enhancement in H3 binding due to phosphorylation.

The formation of transcriptionally silent chromatin, so called heterochromatin, is critical for genomic stability and cell differentiation^1^,^2^,^3^. The higher order structure of heterochromatin is maintained by a nonhistone chromosomal protein, termed heterochromatin protein 1 (HP1)^4^,^5^,^6^. HP1 family proteins contain two functionally distinct globular domains, the N-terminal chromodomain (CD) and the C-terminal chromo-shadow domain (CSD). The CD directly binds to lysine 9 methylated histone H3 (H3K9me), a hallmark of heterochromatin^7^,^8^,^9^, whereas the CSD is responsible for HP1 homodimerization, which mediates the condensation of nucleosomes containing H3K9me^10^. The CD contains an N-terminal three stranded β sheet and a C-terminal α helix that form a globular domain, and binds to H3K9me by a cage formed by three aromatic residues^11^,^12^. For example, in the mouse HP1β CD, tyrosine 20, tryptophan 41 and phenylalanine 44 form the aromatic cage that recognizes the methylated lysine residue of H3K9me^12^.

In mice there are three HP1 isoforms, HP1α, HP1β and HP1γ with highly conserved CDs that are thought to share a similar structural feature to interact with H3K9me. By contrast, the CDs of these isoforms are connected to a less-conserved N-terminal tail, which is hypothesized to contribute to their isoform-specific function^6^. In HP1α, in particular, there are four successive serine residues in the N-terminal tail that are constitutively phosphorylated *in vivo*^13^. While HP1α CD itself can bind to H3K9me, its affinity becomes much stronger when the N-terminal tail is phosphorylated^13^. However, the molecular mechanisms underlying this enhancement remain elusive.

To reveal the role of the phosphorylated tail in HP1 binding to H3K9me, we have compared NMR structures of the CD connected to the N-terminal tail in both its phosphorylated form (phos-NCD) and its un-phosphorylated form (unmod-NCD). The only noticeable effect of phosphorylation on the CD was a change in the dynamics of the N-terminal tail, while the tertiary structure of the structured domain remained intact: that is, the flexibility of the segment containing successive phosphorylated serine residues was significantly reduced as compared with the un-phosphorylated segment. Small angle X-ray scattering (SAXS) experiments revealed that both phos-NCD and unmod-NCD hold an extended string-like structure, but the string of phos-NCD is more extended than that of unmod-NCD. The role of the phosphorylated serine residues in HP1 binding to H3K9me was further confirmed by binding experiments using a series of N-terminal tail truncated CDs with or without phosphorylation. In addition, replica exchange molecular dynamics (REMD) simulations were performed for both phos-NCD and unmod-NCD in their free states and in complex with H3K9me to reveal their characteristic extended string-like structures. REMD enabled exhaustive sampling of the flexible N-terminal tail of NCD and the flexible C-terminal region of the H3K9me peptide interacting with CD in an aqueous environment. We constructed the structural ensemble from 2.4-μs simulations for each of the four simulation systems to illustrate the detailed atomic features of the flexible N-terminal tail. Together, these results uncover a novel structural role of phosphorylation to ensure and specify protein-protein interactions.

## Results and Discussion

### Structures of phos-NCD and unmod-NCD

To examine the structural basis for the role of N-terminal tail phosphorylation in HP1α, we prepared NMR samples of ^13^C-, ^15^N-labeled unmod-NCD and phos-NCD, both of which comprised amino acids 1–80 of HP1α. The phos-NCD sample was prepared by co-expression with casein kinase II. The phosphorylation state of NCD was checked by MALDI-TOF MS, which verified that all four serine residues in HP1α’s N-terminal tail were phosphorylated.

The HSQC spectra of unmod-NCD and phos-NCD showed well resolved NMR signals (Supplementary Fig. S1). Their structures were determined by using distance restraints estimated from NOEs (739 for phos-NCD and 652 for unmod-NCD), hydrogen-bond restraints (18 for phos-NCD and 15 for unmod-NCD), and dihedral restraints (94 for phos-NCD and 98 for unmod-NCD) (Table 1). Notably, for both proteins, all medium- and long-range NOEs and hydrogen bonds were observed only in each CD portion, comprising amino acids 20–80, indicating that neither N-terminal tail region (amino acids 1–19) held tertiary structure.

**Figure 1:**
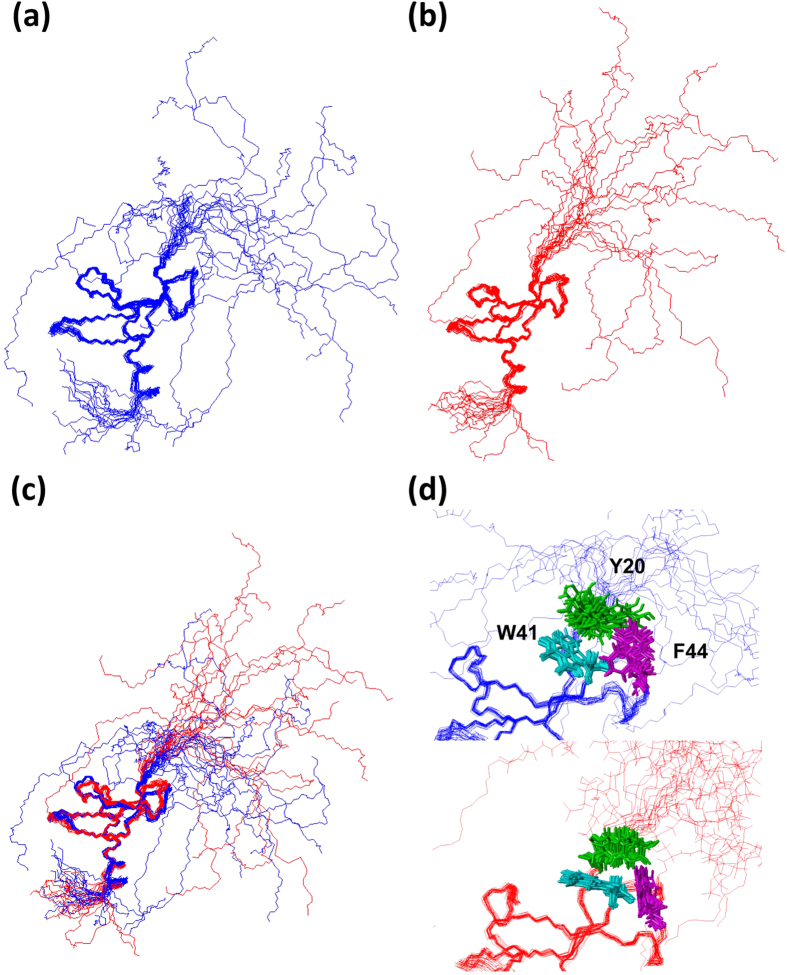
The 20 best calculated NMR structures of unmod-NCD (**a**) and phos-NCD (**b**) of HP1α, and their super positions (**c**). Unmod-NCD is shown in blue and phos-NCD is shown in red. (**d**) Superposition of the aromatic cage formed by Tyr 20, Trp 41 and Phe 44, and backbones of unmod-NCD (top) and phos-NCD (bottom).

The solution structures of both unmod-NCD (Fig. 1a) and phos-NCD (Fig. 1b) are well resolved except for their tails (Supplementary Figs S2 and S3); both CD structures of amino acids 20–67 are well defined with backbone root-mean-square-deviations (RMSDs) of 0.42 ∓  0.10 Å in unmod-NCD and 0.40 ∓ 0.11 Å in phos-NCD, and with heavy atom RMSDs of 1.02 ∓ 0.08 Å in unmod-NCD and 0.92 ∓ 0.11 Å in phos-NCD (Table 1). The CD structures (amino acids, 20–75) of unmod-NCD and phos-NCD, consisting of three β strands and a C-terminal α helix, are well superimposed with an RMSD of 1.43 Å (Fig. 1c), and the aromatic cage structures formed by Tyr 20, Trp 41 and Phe 44 are also well defined (Fig. 1d). It is, therefore, apparent that the CD forms a domain structure independent of the N-terminal tail and irrespective of its phosphorylation state.

Although NMR revealed that both proteins have essentially the same CD structure and a similarly disordered N-terminal tail (Fig. 1c), unmod-NCD seems to adopt a more broadly distributed random N-terminal tail structure as compared with phos-NCD. Regarding the tail structures of unmod-NCD and phos-NCD, distance restraints were obtained only from similar sequential NOEs without medium and long range NOEs. The different distributions of the disordered tail structures were not caused by static NMR structural data, but maybe due to simulated annealing calculations, which included repulsive forces between atoms. The phosphorylated serine residue is more bulky than unphosphorylated serine; as a result, the four phosphorylated serine residues in the N-terminal tail of phos-NCD tend to form a more extended string owing to the successive repulsions of the bulky phosphate groups as compared with the un-phosphorylated tail.

### Dynamics and chemical shift changes of unmod-NCD and phos-NCD

**Figure 2:**
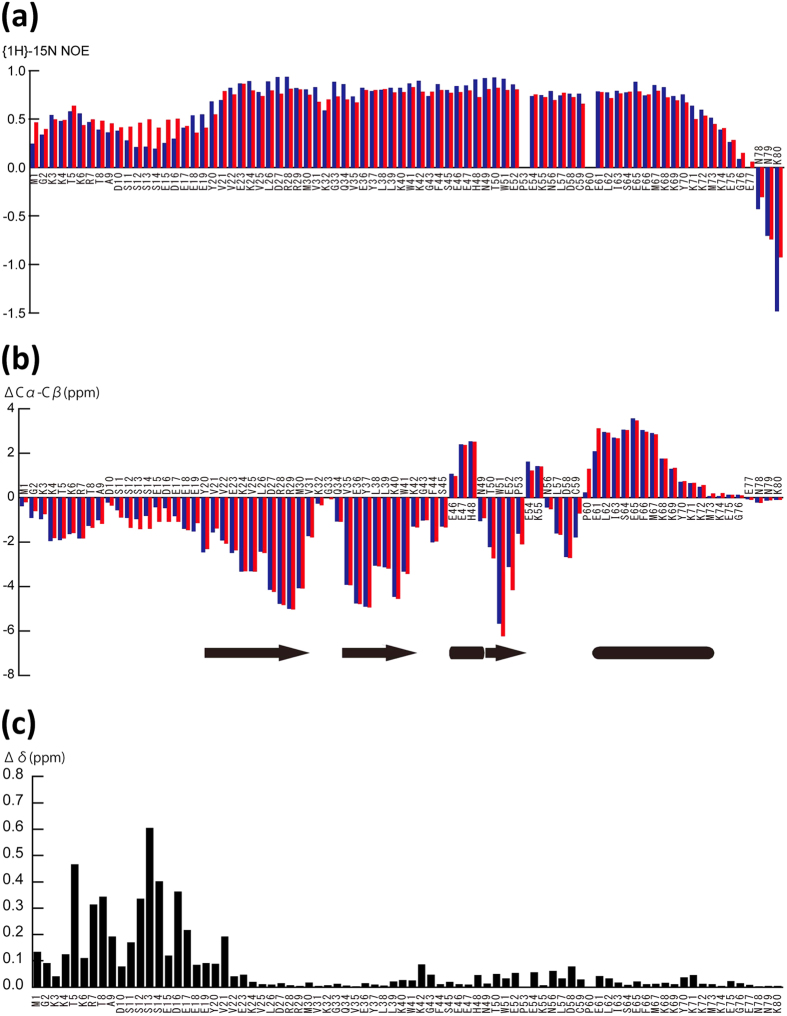
NMR comparison between unmod-NCD and phos-NCD. (**a**) {^1^H}-^15^N hetero NOE values for unmod-NCD (blue bars) and phos-NCD (red bars). (**b**) Chemical shift indices of unmod-NCD (blue bars) and phos-NCD (red bars). The chemical shift index of each residue at i-th position is calculate as ΔCα − Cβ = [{Cα(i − 1) + Cα(i) + Cα(i + 1)} − {Cβ(i − 1) + Cβ(i) + Cβ(i + 1)}]/3. (**c**) Difference in chemical shift between phos-NCD and unmod-NCD. Δδ of ^1^H and ^15^N chemical shifts upon phosphorylation was calculated according to Δδ = {(Δδ ^1^H)^2^ + (Δδ ^15^N/5)^2^}^1/2^.

To compare the dynamic structures of the tails of phos-NCD and unmod-NCD, we examined {^1^H}-^15^N hetero nuclear Overhauser effects (NOEs)^14^,^15^ (Fig. 2a; unmod-NCD, blue bars; phos-NCD, red bars). {^1^H}-^15^N hetero NOE relaxation of backbone ^15^N spins reflects dynamic fluctuations of individual N-H bonds on the pico- to nanosecond scale^14^,^15^. The CD portion (amino acids 20–80) showed essentially the same hetero NOE values in both protein forms: nearly 0.6 for the 20–70 amino acid region with gradually decreasing values for the 71–77 amino acid region, and negative values for the C-terminal 78–80 amino acid region. These data correspond well with the NMR structures, in which both CDs hold a globular rigid structure with a flexible C-terminus. The dynamic character observed for both forms is typical of globular proteins. Both CD portions are dynamically and statistically independent from the phosphorylation state of their N-terminal tails.

Surprisingly, however, both N-terminal tails exhibited positive hetero NOE values for amino acids 1–19 (Fig. 2a), suggesting that they are not as flexible as the C-terminus of the CD. In addition, all amino acids in the phosphorylated tail exhibited relatively high hetero NOE values between 0.4 and 0.5, suggesting that this portion is more likely to be an extended string rather than the entirely flexible random coil observed for the C-terminus.

In unmod-NCD, the region _9_ADSSSSED_16_ had slightly reduced hetero NOE values as compared with phos-NCD, suggesting that the successive unphosphorylated serine residues in the tail behave as a flexible chain as compared with the phosphorylated ones. In unmod-NCD, a basic segment comprising _3_KKTKR_7_ and an acidic segment comprising _15_EDEEE_19_ are connected by this flexible linker, which might enable the segments to dynamically and intra-molecularly interact with each other.

Upon phosphorylation of the four serine residues, the serine and acidic segments form a long negatively charged segment comprising _10_DpSpSpSpSEDEEE_19_, the whole of which behaves like an extended string owing to both a series of electrostatic repulsions between neighboring amino acids and the repulsion between the successive bulky phosphate groups of the serine residues as observed in the NMR structure. In this case, the N-terminal _3_KKTKR_7_ segment might dynamically and intra-molecularly interact with the _10_DpSpSpSpS_14_ segment by means of a presumed short hairpin formed by _8_TA_9_. Thus, the phosphorylated tail of CD would adopt an extended longer structure as compared with the un-phosphorylated tail.

The extended string-like structures of the N-terminal tails were supported by the chemical shift indices of Cα – Cβ (Fig. 2b), which are markers of secondary structure^16^,^17^. Indeed, both unmod-NCD and phos-NCD showed essentially the same pattern of indices for their CD portions, consistent with their determined tertiary structures. As compared with the C-terminal amino acids (73–80), which show no apparent secondary structure, the N-terminal tails in both protein forms are likely to adopt a partially extended structure. Both N-terminal tails have similar patterns of basic and acidic segments; however, the phosphorylated serine segment of _11_pSpSpSpSED_16_ seems to have a significantly more extended conformation as compared with the un-phosphorylated one: in the phosphorylated N-terminal tail, both the basic segment comprising _3_KKTKR_7_ and the phosphorylated serine and acidic segment comprising _11_pSpSpSpSEDEEE_19_ are likely to be partially extended. The chemical shift indices of the N-terminal tail of both unmod-NCD and phos-NCD also correspond well to the proposed string-like structures indicated by the hetero NOE values.

Upon phosphorylation, the chemical environment of the N-terminal tail altered, as shown by the chemical shift changes (Fig. 2c). As indicated by both NMR structures, by contrast, no significant chemical shift changes were observed in the CD portions except for the N-terminal Tyr20 and Val21. In the N-terminal tail, phosphorylation induced great chemical shift changes of the successive serine residues; however, significant chemical shift changes were also observed for Thr5, Arg7, Thr8 and Ala9 in the basic segment, and Asp16 and Glu17 in the acidic segment, suggesting that the chemical environment of each of these segments was significantly altered upon phosphorylation. This is likely to be caused the different interaction of the basic and acidic segments in phos-NCD as compared with unmod-NCD as stated above.

We tried to examine direct interactions between the basic segment _3_KKTKR_7_ and the phosphorylated serine segment _10_DpSpSpSpS_14_ in phos-NCD or the acidic segment of _15_EDEEE_19_ in unmod-NCD by homo-nuclear NOE spectroscopy with several mixing times; however, no interactions were detected. The presumed interaction of the basic segment with the acidic or phosphorylated segment might be too dynamic to show any detectable NOE between them.

In the presumed folded back conformation of unmod-NCD, the basic segment of _3_KKTKR_7_ could potentially also interact with Tyr20 and Val21 in the CD, which neighbor the acidic segment _15_EDEEE_19_, because both amino acids exhibited significant chemical shift changes upon phosphorylation (Fig. 2c). Both Tyr20 and Val21 also exhibited substantial chemical shift changes upon binding to the H3K9me peptide (see below). For unmod-NCD, therefore, the intra-molecular interaction between the basic segment _3_KKTKR_7_ and the acidic segment plus Tyr20 and Val21 is likely to mimic the inter-molecular interaction between the H3K9me peptide and the acidic segment plus Tyr20 and Val21.

It might be possible that the differences in chemical shift observed between unmod-NCD and phos-NCD originate from different dimer or multimer associations due to their N-terminal tails. We checked the HSQC spectra of 10-fold diluted samples of both proteins, but no significant signal changes were observed.

### Small angle X-ray scattering (SAXS) of CD, unmod-NCD and phos-NCD

**Figure 3:**
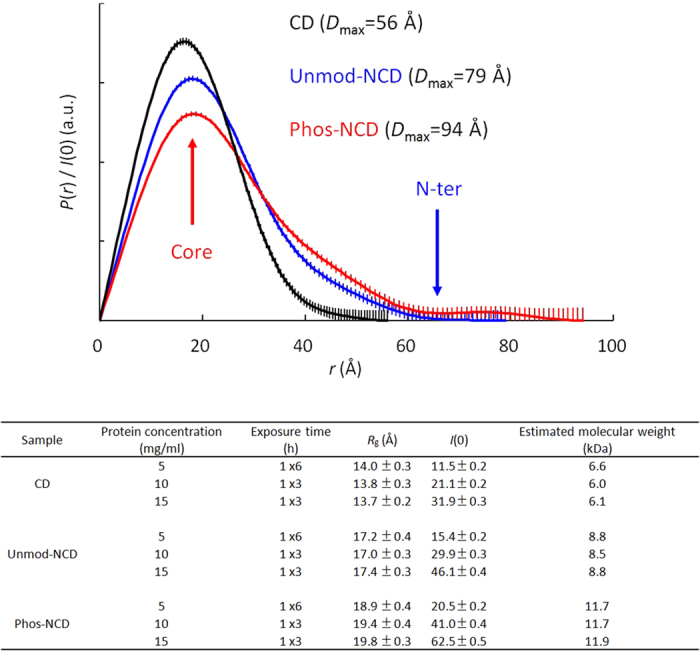
*P*(*r*) functions for CD (black), unmod-NCD (blue) and phos-NCD (red) (top) and the radius of gyration *R*_g_ and estimated molecular weight from the forward scattering intensity *I*(0), for CD, unmod-NCD and phos-NCD (bottom).

To confirm the structural differences between unmod-NCD and phos-NCD, small angle X-ray scattering (SAXS) experiments were carried out on CD, unmod-NCD, and phos-NCD. To check inter-particle interference, SAXS data were collect at three different protein concentrations, 5, 10 and 15 mg/ml (Supplementary Fig. S4). The radius of gyration *R*_g_, and the estimated molecular weight from the forward scattering intensity *I*(0), of CD, unmod-NCD, and phos-NCD are summarized in Fig. 3. For all three proteins, *R*_g_ did not depend on protein concentration, indicating that there was no apparent inter-particle interference in the SAXS data. The estimated molecular weight of CD, unmod-NCD, and phos-NCD was close to the exact Mw of 7.6, 9.8, and 10.1 kDa, respectively, indicating that all proteins existed as a monomer and the data did not contain scattering from aggregated protein. The *R*_g_ value of the CD at the three different protein concentrations was estimated as 14.0 ± 0.3, 13.8 ± 0.3 and 13.7 ± 0.2 Å, which are close to the *R*_g_ value calculated from the NMR structure of the CD (15.0 Å). This indicates that the structure of CD is globular, as observed for the NMR structure.

We converted the scattering profiles at 5 mg/ml to the *P*(*r*) function and the estimated maximum particle dimension, *D*_max_ (Fig. 3). For the CD, the *P*(*r*) function showed a single peak corresponding to the pair distribution of atoms within a globular protein. On the other hand, the *P*(*r*) function of both unmod-NCD and phos-NCD showed an extended tail that was not observed for the CD. The *D*_max_ of unmod-NCD (79 Å) and phos-NCD (94 Å) was larger than that of the CD (56 Å). The extended tail of the *P*(*r*) function and larger *D*_max_ for unmod-NCD and phos-NCD is due to scattering from the N-terminal tail region in each case. Further comparison showed that the extended tail of *P*(*r*) function of phos-NCD was more extended than that of unmod-NCD, and *R*_g_ and *D*_max_ of phos-NCD were larger than those of unmod-NCD. These data indicate that the conformation of the N-terminal region of phos-NCD is more extended than that of unmod-NCD.

Notably, the *P*(*r*) functions calculated from the NMR structures predicted *D*_max_ value of 82 Å for unmod-NCD and 107 Å for phos-NCD (Supplementary Fig. S5). The NMR structures gave a larger *D*_max_ for phos-NCD than for numod-NCD, which corresponded well qualitatively with the SAXS data; however, the magnitudes of *D*_max_ derived from the NMR structures were larger than the observed SAXS values. This suggests that the N-terminal tail structure of both unmod-NCD and phos-NCD in solution is more compact than that in the static NMR structures. Comparison of the static NMR structures of the two N-terminal tails reflects only the bulkiness of the phosphate groups in phos-NCD.

### Binding between H3K9me and N-terminally truncated CDs

**Figure 4:**
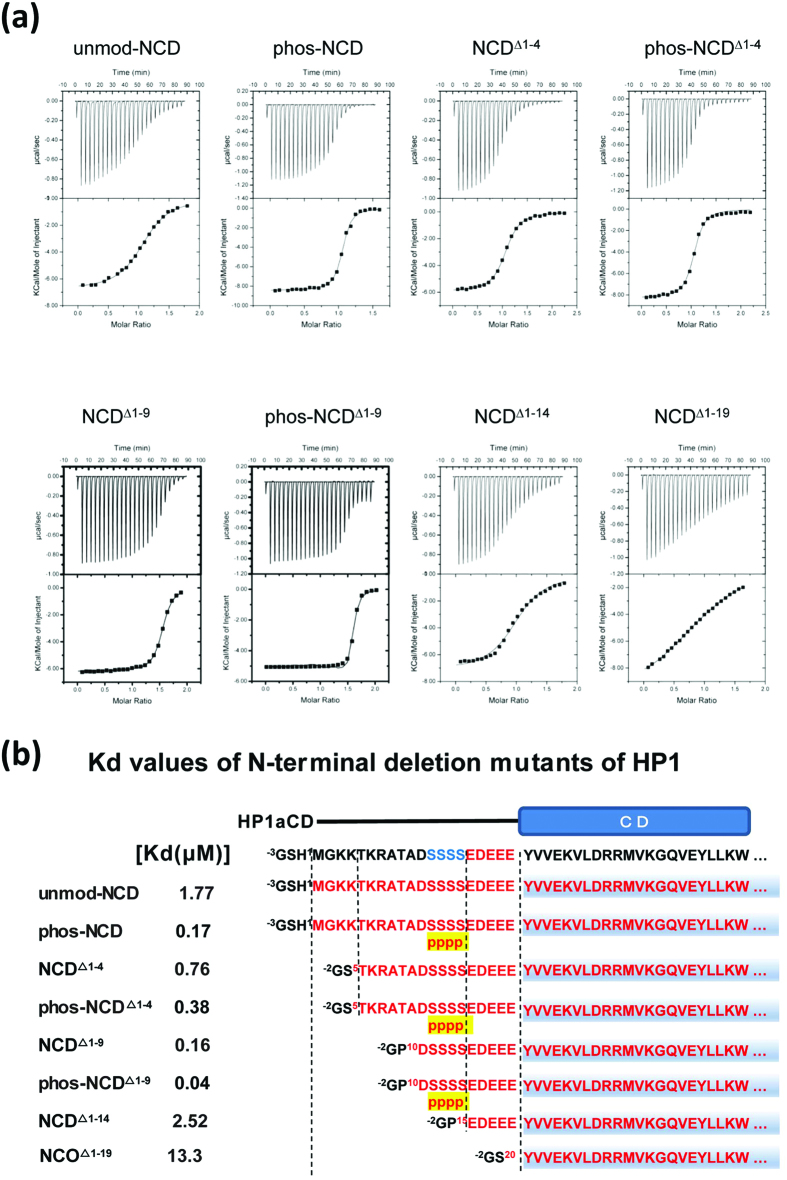
Binding affinities of various N-terminal deleted mutants of unmod-NCD and phos-NCD by ITC experiments. (**a**) Upper panels: raw data for heat measured upon injection of the H3K9me peptide into each protein. Lower panels: integrated heat of injections. The solid line shows the best fit of the data. (**b**) Schematic view showing the deletion mutants together with their Kd values.

To confirm the extended string-like structure together with the folded back dynamic structures of the N-terminal tail of both unmod-NCD and phos-NCD, we examined the effect of N-terminal deletion on the binding of NCD to a H3K9me peptide (22-mer, comprising amino acids 1–21 of histone H3 with a C-terminal tyrosine for quantitative analysis), by using isothermal calorimetry (ITC) experiments (Fig. 4, Supplementary Fig. S6). phos-NCD, comprising amino acids 1–80 of HP1α, bound to H3K9me with approximately 10-fold stronger affinity (Kd = 0.17 μM) than unmod-NCD (Kd = 1.77 μM). Systematic deletion mutants showed that the CD domain alone, NCD^Δ1–19^ (amino acids 20–80), had the weakest binding activity (Kd = 13.3 μM) suggesting that the N-terminal tail mediates binding between H3K9me and CD. Addition of the acidic segment to the CD, NCD^Δ1–14^ (amino acids 15–80), led to about 5-fold stronger activity than the CD alone, and further addition of the serine segment, NCD^Δ1–9^ (amino acid 10–80) resulted in about 83-fold stronger binding. Notably, the phosphorylated NCD^Δ1–9^ form, phos-NCD^Δ1–9^, showed the strongest binding of all mutants (Kd = 40 nM) with about 330-fold stronger activity than the CD. These data suggest that the acidic segment _15_EDEEE_19_ together with the phosphorylated _10_DpSpSpSpS_14_ region is responsible for enhancing the interaction with the H3K9me peptide, _*1*_*ARTKQTAR*(*Kme*)*STGGKAPRKQLA*_*21*_(*Y*), (hereafter to clarify *italic* font is used for the amino acids of H3, and normal font for HP1), where *Kme9* fits into the aromatic cage formed by Tyr20, Trp41 and Phe44 and the _*8*_*R*(*Kme*)*STGGKAPRK*_*18*_ segment of the histone H3 tail seems to interact with the _10_DpSpSpSpSEDEEE_19_ segment in the phos-NCD tail.

As compared with NCD^Δ1–9^, NCD^Δ1–4^ (amino acids 5–80) containing five additional amino acids, _5_TKRTA_9_, led to about 4-fold weaker binding to H3K9me, suggesting that the two amino acids _6_KR_7_ might intra-molecularly bind to the acidic _15_EDEEE_19_ segment, thereby inhibiting interactions with the _*8*_*R*(*Kme*)*STGGKAPRK*_*18*_ segment of the H3K9me peptide. However, when the serine residues of NCD^Δ1–4^ were phosphorylated (phos- NCD^Δ1–4^), binding to H3K9me was recovered to a value similar to that of un-phosphorylated NCD^Δ1–9^. As compared with NCD^Δ1–9^, the basic segment _3_KKTKR_7_ in numod-NCD might intra-molecularly bind more strongly to the acidic string _15_EDEEE_19_, causing 6-fold weaker binding to the H3K9me peptide. In phos-NCD, however, the phosphorylated serine residues would stiffen the string, thereby inhibiting the folded-back interaction of the basic string _3_KKTKR_7_ with the acidic string _15_EDEEE_19_. Thus, the observed order of binding affinity (Kd) was phos- NCD^Δ1–9^ (0.04 μM) ≫ phos-NCD (0.17 μM) ~ NCD^Δ1–9^ (0.16 μM) ~ phos- NCD^Δ1–4^ (0.38 μM) > NCD^Δ1–4^ (0.76 μM) >unmod-NCD (1.77 μM) ~ NCD^Δ1–14^ (2.52 μM) ≫ CD (13.3 μM).

### Comparison of CD, unmod-NCD and phos-NCD bound to H3K9me peptide

**Figure 5:**
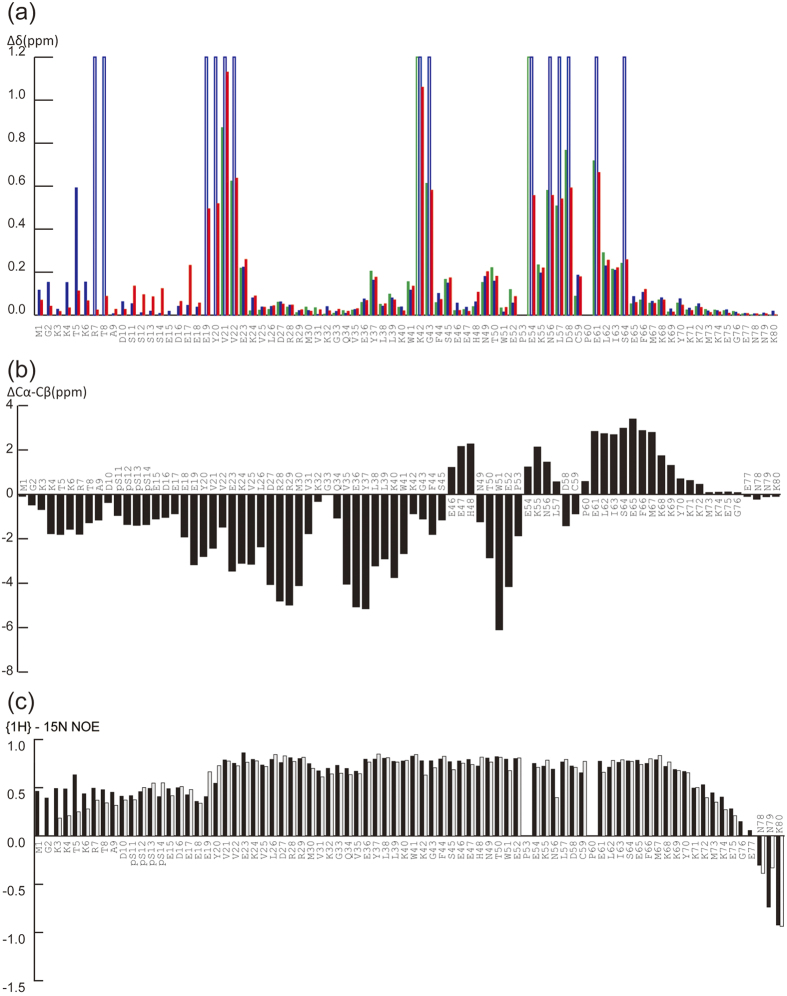
Chemical shift changes, chemical shift indices and {^1^H}-^15^N hetero NOE values. (**a**) Changes in ^1^H and ^15^N chemical shifts of CD (green), unmod-NCD (blue) and phos-NCD (red) upon binding to the H3K9me peptide according to Δδ = {(Δδ ^1^H)^2^ + (Δδ ^15^N/5)^2^}^1/2^, where signals that are disappeared after binding to H3K9me are indicated as open blue (unmod-NCD) or open green (CD) bars over 1.2 ppm. (**b**) Chemical shift indices of the complex of phos-NCD bound to H3K9me peptide, where the chemical shift index of each residue at i-th position is calculated as ΔCα − Cβ = [{Cα(i − 1) + Cα(i) + Cα(i + 1)} − {Cβ(i − 1) + Cβ(i) + Cβ(i + 1)}]/3. (**c**) {^1^H}-^15^N hetero NOE values for phos-NCD in its free state (black bars) and its complex with H3K9me (gray bars).

Next, we examined the structural changes that occur in CD, unmod-NCD, and phos-NCD upon binding to H3K9me peptide by NMR (Fig. 5). Notably, the isolated CD and the CD region in phos-NCD showed essentially the same chemical shift changes upon binding to H3K9me peptides (Fig. 5a), although the binding affinities differed considerably: phos-NCD bound H3K9me about 80 times more strongly than the CD. This suggests that the structure of the CD portion in phos-NCD bound to H3K9me is not altered by the presence of the phosphorylated tail; in other words, the tail does not interfere with the CD but enhances its interaction with the H3K9me peptide. In addition, Glu19 and Tyr20 showed substantial chemical shift changes after binding to H3K9me peptide (Fig. 5a), indicating that these two residues contribute to the interaction with _*8*_*R*(*Kme*)_*9*_ of H3, as will be shown below in the complex structure.

The chemical shift changes in the N-terminal tail of phos-NCD on binding to H3K9me were small; however, as compared with the changes of the C-terminal portion, significant changes were observed for the four phosphorylated serine residues and Asp16 and Glu17 in addition to Glu19 (Fig. 5a). These residues are probably responsible for the interaction with the _*8*_*R*(*Kme*)*STGGKAPRK*_*18*_ segment of H3K9me.

As compared with CD and phos-NCD, unmod-NCD showed greater spectral changes upon binding to H3K9me (Fig. 5a). On formation of the complex of unmod-NCD and H3K9me, many NMR signals in the tail region in addition to the CD region disappeared, as indicated by open blue bars. The broadening and disappearance of signals may be caused by conformational fluctuations upon binding to H3K9me; such fluctuations were not observed in the complexes of CD and phos-NCD with H3K9me. Strikingly, the signals of Arg7 and Thr8 in unmod-NCD disappeared upon binding to H3K9me (Fig. 5a), suggesting that the conformations of these two amino acids fluctuate in the complex. As observed in the experiments with tail deletion mutants, the basic segment _3_KKTKR_7_ seems likely to interact with the acidic segment _15_EDEEE_19_ plus Tyr20 and Val21 in the un-phosphorylated tail. Upon binding to H3K9me, the acidic _15_EDEEE_19_ segment plus Tyr20 and Val21 might interact dynamically with both the H3K9me basic segment _*8*_*R*(*Kme*)*STGGKAPRK*_*18*_ and the basic _3_KKTKR_7_ segment of unmod-NCD. In the case of unmod-NCD, even though *Kme9* of H3 is specifically captured in the aromatic cage formed by Tyr20, Trp41, and Phe44 of the CD portion, binding of the backbone of the _*8*_*RKmeSTGGKAPKR*_*18*_ segment to the acidic _15_EDEEE_19_ segment plus Tyr20 and Val21, seems to be dynamically inhibited by intra-molecular binding of the basic segment _3_KKTKRT_8_. This interference might lead to disappearance of the signals for Arg8, Thr9, Glu19, and Tyr20 in unmod-NCD upon binding to the H3K9me peptide. Partial binding of the basic segment of unmod-NCD to the acidic segment would cause changes in the complex structure of unmod-NCD and H3K9me in the CD portion. Consistent with this, we obtained broadened signals for amino acids in the H3K9me binding sites of the CD portion, including Val21, Val22, Lys42, Gly43, Glu54, Lys55, Asn56, Leu57, Asp58, Cys59, Glu61, and Ser64 in addition to Tyr20. Indeed, the amino acids with signals that disappeared corresponded well with those that showed significant chemical shift changes in CD and phos-NCD upon binding to the H3K9me peptide.

### NMR of phos-NCD bound to H3K9me peptide

The chemical shift indices of phos-NCD bound to H3K9me remained essentially same as those of free phos-NCD (Fig. 5b), indicating that the secondary structures of phos-NCD did not change upon binding to H3K9me. However, small but significant differences in chemical shift indices were observed in two regions: namely, _19_EYVV_22_ and _56_NKD_58_. As described in the complex structure below, both regions are involved in interacting with the H3K9me peptide: the backbone of _*5*_*QTARKme*_*9*_ of H3 is sandwiched between the _19_EYVV_22_ and _56_NKD_58_ backbones of the CD portion in phos-NCD.

In the complex of phos-NCD bound to H3K9me, almost all portions except two regions, _3_KKTKRT_8_ and _19_EY_20_, showed essentially the same hetero NOE as free phos-NCD (Fig. 5c). In the first case, the basic _3_KKTKR_7_ segment is probably inhibited from interacting with the phosphorylated _10_DpSpSpSpS_14_ segment by the _*8*_*R*(*Kme*)*STGGKAPRK*_*18*_segment of the histone H3 tail, so it is now more freely exposed to solvent; as a result, the flexibility of the basic _3_KKTKRT_8_ segment seems to be lowered. In the second case, the flexibilities of Glu19 and Tyr20 region are reduced by binding to H3K9me. As shown below, the backbone of the Glu19 and Tyr20 portion is stabilized by hydrogen bonding to the backbone of _*5*_*QTARKme*_*9*_ in H3K9me; thus, the two amino acids are stiffened by binding.

### Structural analysis of phos-NCD bound to H3K9me peptide

To determine the tertiary structure of phos-NCD bound to the H3K9me peptide, we prepared an NMR sample of a complex formed between ^13^C-, ^15^N-labeled phos-NCD (residues 1–80) of HP1α phosphorylated at serine residues 11–14 and the unlabeled H3K9me peptide (residues 1–18) of histone H3. A total of 637 distance restraints estimated from NOEs, 7 hydrogen-bond restraints (for phos-NCD), 99 dihedral restraints (for phos-NCD) and 43 intermolecular NOEs were used to determine the structure (Table 1).

**Figure 6:**
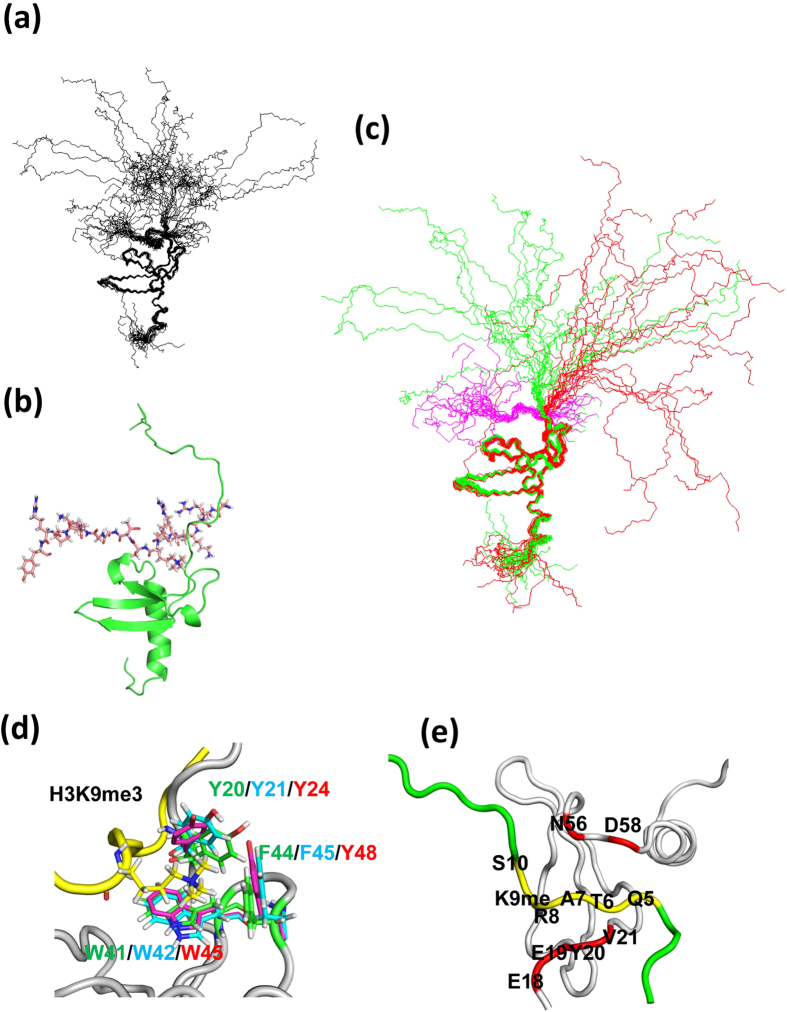
Solution structures of phos-NCD bound to the H3K9me peptide. (**a**) Superposition of the 20 best NMR structures. (**b**) Lowest energy structure of the complex. phos-NCD is shown as a green ribbon; the H3K9me is shown as a magenta stick. (**c**) Structural comparison between unbound phos-NCD (red) phos-NCD (green) bound to the H3K9me peptide (magenta).

The NMR structures of phos-NCD bound to the H3K9me peptide were well defined (Fig. 6a, Table 1, Supplementary Fig. S7). Figure 6c shows superposition of the structures of free phos-NCD (red) and phos-NCD (green) bound to H3K9me (magenta). Upon binding to H3K9me, the structure of the CD moiety is not altered as indicated by the RMSD value of 0.97 Å between the two structures. The three methyl groups of H3K9 are surrounded by three aromatic residues, Tyr20, Trp41 and Phe44 of phos-NCD and the aromatic cage structure in the complex is essentially the same as that in phos-NCD, *Drosophila* HP1a, and mouse HP1β (Supplementary Fig. S8).

The backbone of _*5*_*QTARKS*_*10*_ in the H3K9me peptide interacts with two backbone strands of phos-NCD (Supplementary Fig. S8): the Asp58 and Asn56 strand; and the Glu18, Glu19, Tyr20 and Val21 strand. These amino acids of phos-NCD showed significant chemical shift changes upon binding to H3K9me. The mode of backbone interaction is very similar to that observed between the *Drosophila* CD and the H3K9me peptide. In the present NMR structure determination, there were no direct interactions such as homo-nuclear NOEs between the phosphorylated HP1 tail and the histone H3 tail. However, two presumed interacting structures were obtained (Supplementary Fig. S8): one shows interactions between *Arg8* of the H3K9me peptide and phosphorylated Ser14 of phos-NCD, together with *Arg17* of the H3K9me peptide and phosphorylated Ser11 of phos-NCD; the other shows interactions between *Arg8* of the H3K9me peptide and Glu15 of phos-NCD, together with *Arg17* of the H3K9me peptide and Asp10 of phos-NCD.

### REMD simulations of unmod-NCD and phos-NCD, with and without the H3K9me peptide

To provide more detailed structures to interpret the results obtained from the NMR and SAXS experiments, we generated structural ensembles for the four different systems-i.e., the complex forms of unmod-NCD and phos-NCD, and the unbound forms of unmod-NCD and phos-NCD, by replica exchange molecular dynamics (REMD) simulations. For each system, 48 replicas were sufficient to connect each ensemble at 290.53 K to that at 443.15 K, and the potential energy distributions significantly overlapped between neighboring replicas to give an average exchange rate of 0.1 (Supplementary Fig. S9). During a simulation time of 50 ns, each replica went from the lowest to the highest temperatures several times, yielding a well converged structural ensemble at 293.15 K (the second lowest temperature among the 48 replicas; Supplementary Fig. S9).

During the REMD simulations, the core region of CD (20–67) maintained its structure with a root mean square fluctuations (RMSFs) for Cα atoms of 1.15, 1.15, 0.75 and 0.73 Å for unmod-NCD and phos-NCD without the H3K9me peptide, and unmod-NCD and phos-NCD in complex with peptide, respectively. Although phosphorylation of the N-terminal serine residues scarcely affected the fluctuations, formation of the complex significantly stiffened the core region. The H3K9me peptide connects the N-terminal (residues 17–20) and the C-terminal (residues 55–58) regions, and stabilizes the flexible loop via interactions with the aromatic cage (residues 20, 41, and 44). The Cα RMSFs of the C-terminus after fitting the core region were 9.19, 8.81, 5.10 and 5.39Å, respectively, for the systems in the above order, indicating the intrinsically flexible structure of the C-terminus. The differences between the unbound and peptide-bound structures may simply reflect the difference in flexibility of the core regions. On the other hand, the Cα RMSFs of the N-terminal tail strongly depended on both the phosphorylation state and the binding of the H3K9me peptide (10.78, 12.69, 7.62 and 5.39 Å). These observations agree with the results from our experiments. In the unbound state, phosphorylation tends to make the N-terminal tail more extended, corresponding to the increase in the radius of gyration observed in the SAXS experiments. In contrast, peptide binding reduces the RMSFs more on phos-NCD, indicating stronger interactions between the N-terminal tail and the H3K9me peptide.

In terms of the behavior of the flexible N-terminal tail, the structural ensembles obtained from the REMD simulations for unmod-NCD and phos-NCD in their free states were compared. The distribution of the far N-terminus (C_α_ atom of Met1) was enhanced by phosphorylation of the N-terminal serine residues (Supplementary Fig. S9d). This was also observed in the statistical analysis in Supplementary Fig. S9e, where the distribution of the minimum distance between the C_α_ atoms of residue 1–10 (the edge of the N-terminal tail) and those of residues 19–21/56–59 (the atoms that form contact with the H3K9me peptide in the complex form) was calculated for unmod-NCD and phos-NCD. Unmod-NCD has a high frequency of contacts between the two regions, whereas phos-NCD exhibits a lower frequency of contacts. This indicates that phosphorylation breaks favorable interactions between the N-terminal tail and the core region, and thus distributes the N-terminal tail over a wider range of space. Supplementary Fig. S10 shows ten representative structures of unmod-NCD and phos-NCD, which are extremely flexible but appear to contain the non-specific electrostatic interactions between the basic (3–8) and acidic (15–19) segments in unmod-NCD, and the basic (3–8) and phosphorylated serine and acidic (10–19) segments in phos-NCD.

**Figure 7:**
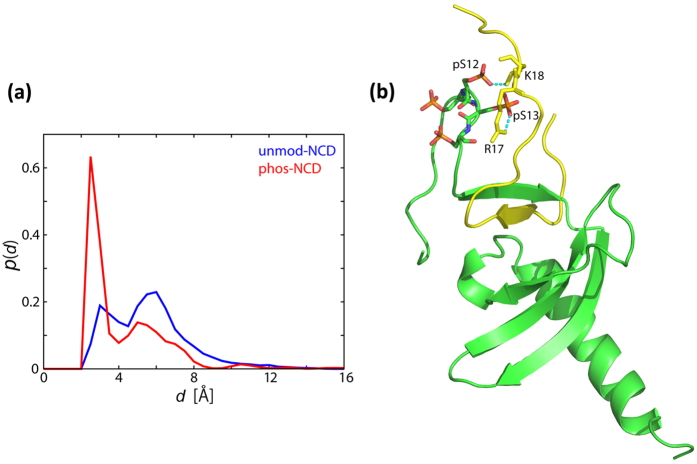
Results of REMD simulations for unmod-NCD, and phos-NCD bound to the H3K9me peptide. (**a**) Probability distributions of the minimum distance between the C_α_ atoms of *K14/R17/K18* of the H3K9me peptide (the basic residues at the C-terminal after K9me) and those of the four serines (11–14) of NCD, for unmod-NCD (blue) and phos-NCD (red) in the REMD simulation of the bound state. (**b**) Representative structure of phos-NCD complexed with H3K9me obtained in the REMD simulation of the bound state. Ionic interactions between pS12 and *K18*, and pS13 and *R17* are indicated by dotted cyan lines.

REMD simulations demonstrated that, in the H3K9me peptide-bound state, the phosphorylated _11_pSpSpSpS_14_ segment in phos-NCD showed strong electrostatic interactions with the basic residues *Lys14, Arg17*, and *Lys18* in the H3K9me peptide. Figure 7a clearly shows that the phosphorylation strongly enhanced the interactions between the N-terminal tail and the histone tail. Figure 7b is a snapshot of the REMD simulation for phos-NCD bound to the H3K9me peptide, exhibiting salt bridges between phosphorylated Ser12 and *Lys18* and between phosphorylated Ser13 and *Arg17*.

## Conclusion

The recognition mode of the chromodomains of HP1 family proteins for histone H3K9me has been described in detail. A cage formed by three aromatic amino acids—for example, Tyr24, Trp45, and Tyr48 in *Drosophila* HP1a^11^; and Tyr20, Trp41, and Phe44 in mouse HP1β chromodomains^12^—is responsible for capturing the methyl moiety on lysine 9 in the methylated histone H3 tail, and the backbone of the N-terminal histone tail of amino acids 5–8 is sandwiched by two β strands in the chromodomains^11^,^12^. In this context, the structure of HP1α phos-NCD bound to the H3K9me peptide shows a similar binding mode with an aromatic cage formed by Tyr20, Trp41, and Phe44. However, these features do not explain the enhancement in affinity due to phosphorylation of the N-terminal tail of HP1α. Here, we have demonstrated that the HP1α tail segment containing four phosphorylated serine residues _10_DpSpSpSpS_14_, together with the acidic amino acids _15_EDEEE_19_, behave like a negative extended string to interact with the positive amino acids _*8*_*RKmeSTGGKAPRK*_*18*_ in the tail of histone H3. Systematic truncation of the N-terminal tail of numod-NCD and phos-NCD suggested that the CD protein with an entirely negative _10_DpSpSpSpSEDEEE_19_ string-like tail has the strongest affinity for the H3K9me peptide, binding more than 300 times more tightly as compared with the isolated CD alone. These ten negative amino acids seem to adopt a negatively charged extended string that can directly interact with the positively charged histone H3 tail containing K9me.

This mode of interaction between two isolated extended string-like proteins is rather peculiar in the sense of currently known protein-protein interactions. It is well known that proteins that are intrinsically disordered in the unbound state form a rigid structure on their target proteins by a coupled folding and binding mechanism^18^,^19^,^20^,^21^,^22^. Depending on the protein’s amino acid sequences, the folded structures on the target protein is polymorphic, occurring as an amphipathic helix^23^,^24^ or an extended strand^25^,^26^,^27^; however, the complex overall adopts a globular structure.

In the case of the interaction between the tail of phos-NCD and the histone H3 tail, two extended string-like interactions have a dominant effect on binding activity; however, no rigid complex globular structure was observed for these strings. Even in the interaction between the two tails, each tail seemed to retain a dynamic character. It is likely that the two fluctuating strings are dynamically interacting with each other. This dynamic string-like behavior forms the basis of the enhancement in interaction between HP1α and histone H3 resulting from phosphorylation of the tail of the HP1α chromodomain.

## Methods

### Purification of CD without and with the phosphorylated or un-phosphorylated tail

Recombinant CDs without and with the phosphorylated or un-phosphorylated tail were prepared according to a previously described method^13^. In our previous study, phos-NCD was confirmed by LC-MS/MS to contain only phosphorylation of Ser11, Ser12, Ser13 and Ser14. Proteins were expressed in *Escherichia coli* strain BL21 (DE3) star containing the expression plasmid pCold/Amp (HP1αCD) with or without pRSFduet/Kan (CK2) grown in LB medium. Each of the ^15^N-labeled or ^13^C/^15^N-labeled proteins was expressed in M9 minimal medium containing ^15^N-ammonium chloride with or without ^13^C-glucose.

The harvested cells were re-suspended in Buffer A (50 mM Na phosphate buffer (pH 7.0), 500 mM NaCl, 10% glycerol, 1 mM 2-Mercaptoethanol), lysed on ice by sonication and centrifuged. The supernatant of each protein solution was then applied to Ni-NTA super flow (Qiagen) equilibrated with Buffer B (50 mM Na phosphate buffer (pH 7.0), 1 M NaCl, 30 mM imidazole, 1 mM 2-Mercaptoethanol), and each His-tagged sample was eluted by Buffer C (50 mM Na phosphate buffer (pH 7.0), 1 M NaCl, 1 mM 2-Mercaptoethanol ,400 mM imidazole). Each eluted His-tagged sample was dialyzed against Buffer D (50 mM Na phosphate buffer (pH 7.0), 300 mM NaCl, 1 mM 2-Mercaptoethanol) and digested with Thrombin protease at 4 °C overnight. The protein solution was again loaded onto the Ni-NTA column. Fractions passing through the column were concentrated and loaded on HiLoad 26/60 Superdex 75pg against Buffer E (50 mM Na phosphate buffer (pH 7.0), 500 mM NaCl, 1 mM 2-Mercaptoethanol]. Fractions were collected and dialyzed against Buffer F (20 mM KPB (pH 6.8), 10 mM NaCl, 10 or 100% D_2_O, 5 mM DTT). Each sample was checked by mass spectrometry, using a MALDI-TOF Autoflex^TM^ (Bruker Daltonis) to confirm that the modification state of phos-NCD contained four phosphorylated residues. In addition, the HSQC spectra of unmod-NCD and phos-NCD showed that only Ser11, Ser12, Ser13 and Ser14 are typically downshifted by direct phosphorylation^17^, whereas Thr5 and Thr8 are upshifted, indicating that neither threonine residues was directly phosphorylated but both signals were indirectly changed due to the phosphorylation of the four serine residues (Supplementary Fig. S11).

### Preparation of CD with an N-terminally truncated tail

Plasmids encoding the CD with a truncated tail were obtained by PCR using plasmid pCold/Amp (HP1αCD) as a template with appropriate primer sets and Prime Star® Max DNA polymerase (Takara Bio.). Protein expression and purification were performed as described above with small modifications. For protease digestion, each sample was concentrated by Millipore Amicon®Ultra MWCO 5,000 and cleaved by Thrombin at room temperature for 2days in 50mM Na phosphate buffer (pH 7.0), 300 mM NaCl, 1 mM 2-Mercaptoethanol. Each sample was checked by mass spectrometry using a MALDI-TOF Autoflex^TM^ (Bruker Daltonis) to confirm its modification state. During preparation of the phosphorylated D10 mutant, the sample obtained was 5 amino acids longer than expected; thus, the Thrombin recognition site in the vector was changed to an HRV3C recognition site to produce phosphorylated and un-phosphorylated D10 with two amino acids, GP, attached to the N-terminus. All other samples contained three amino acids, GSH, at their N-terminus after cleavage by Thrombin.

### NMR spectroscopy

The protein concentrations were 0.5 mM in 20 mM KPB (pH 6.8), 10 mM NaCl, 5 mM d-DTT and 10% or 100% D_2_O. The NMR experiments were performed at 25 °C on a Bruker Avance 600 MHz spectrometer and 800 MHz spectrometer, both with a 5-mm triple-resonance pulsed-field gradient cryoprobe. Chemical shifts were referenced to the chemical shift of 2,2-dimethyl-2-silapentane-5-sulfonate. The ^15^N and^13^C chemical shifts were referenced indirectly to 2,2-dimethyl-2-silapentane-5-sulfonate using the absolute frequency ratios.

Backbone and side chain resonances were assigned by the following experiments: 2D ^1^H–^15^N HSQC and ^13^C HSQC; 3D HNCO, 3D HN(CO)CA, 3D HNCA, 3D HNCACB, 3D CBCA(CO)NH, 3D HBHA(CO)NH, 3D HCCH-TOCSY, 3D HCCH-COSY, 2D (HB)CB(CGCD)HD, and 2D (HB)CB(CGCDCE)HE; and 3D ^15^N NOESY-HSQC (100 ms) and 3D ^13^C NOESY-HSQC (100 ms). For the complex, intramolecular distance restraints were obtained from 3D ^15^N and ^13^C NOESY–HSQC spectra. Resonance assignments and intramolecular distance restraints for the unlabeled H3K9me3 peptide were obtained from 2D NOESY and TOCSY with a ^13^C-filtered or ^13^C/^15^N filtered pulse scheme^28^. Intermolecular distance restraints were obtained from 3D ^13^C/^15^N X-filtered NOESY spectra^29^. The NOE mixing time in all NOESY experiments was set to 180 ms. All NMR spectra were processed by using the program NMRPipe^30^ and analyzed using the program Olivia (M. Yokochi, S. Sekiguchi, & F. Inagaki, Hokkaido University, Sapporo, Japan) with angle restraints determined by TALOS+ and structures calculated by CYANA v2.1^31^,^32^. CYANA was used to compute seven cycles, each with 600 structures. Each conformer was subjected to 10,000 steps of torsion angle dynamics per cycle. Ultimately, the 20 lowest energy structures were selected to represent each structure. Ramachandran plot statistics for the structures were calculated by using PROCHECK-NMR^33^. The statistics of the structures are summarized in Table 1. The structures were visualized by using MOLMOL^34^ and PyMOL (DeLano, W. L. The PyMOL Molecular Graphics System, http://www.pymol.org).

### Preparation of histone H3 peptides

All of the histone H3 peptides used in this study were purchased from Sigma Genosys.

### Isothermal titration calorimeter (ITC) experiments

Protein solution (40 μM) was loaded into a VP-ITC isothermal titration calorimeter (Microcal, INC.) cell (active cell volume 1.4 ml). The solution was titrated against 400 μM ligand solution via a 250 μl titration syringe. Experiments were carried out at 20 °C. The ligand solution was prepared in the same buffer as the protein (20 mM KPB (pH 6.80), 10 mM NaCl). The heat of dilution generated by the ligand was subtracted, and the binding isotherms were fitted to a one-site binding model by using Origin 7 Software (Microcal, INC.). From the values of *Kd* and Δ*H*, the thermodynamic parameters, Δ*G* and Δ*S* were calculated according to the basic thermodynamic equations;


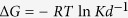



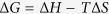


### Small angle X-ray scattering (SAXS)

SAXS measurements were performed at 20 °C with a MicroMax007HF X-ray generator (Rigaku). A PILATUS100K detector (DECTRIS) at a distance of 561 mm from the sample was used to measure scattering intensities. Each sample solution was transferred to a quartz-window cell with a 1 mm path length. Circular averaging of the scattering intensities was carried out to obtain one-dimensional scattering data *I*(*q*) as a function of *q* (*q* = 4πsin*θ*/λ, where 2*θ* is the scattering angle and λ is the X-ray wavelength 1.5418 Å). To correct for interparticle interference, *I*(*q*) data were collected at three different protein concentrations (5, 10, and 15 mg/ml in 50 mM KPB (pH 6.8), 500 mM NaCl, and 5 mM DTT.). The total exposure times were 6 hours for 5 mg/ml, and 3 hours for both 10mg/ml and 15mg/ml. Because the intensity profile did not indicate a concentration effect, the correction for interparticle interference was not applied. To estimate the molecular weight of samples, *I*(*q*) data were collected for hen egg white lysozyme (5.2 mg/ml in 50 mM KPB (pH 6.8), 500 mM NaCl). The data were processed by using the software applications embedded in the ATSAS package [ http://www.embl-hamburg.de/biosaxs/software.html]. The radius of gyration *R*_g_ and forward scattering intensity *I*(0) were estimated from the Guinier plot^35^ of *I*(*q*) in the smaller angle region of *qR*_g_ < 1.3. The distance distribution function *P*(*r*) was calculated in the program GNOM^36^, where the experimental *I*(*q*) data were used in a *q*-range from 0.031 to 0.250 Å^−1^. The maximum particle dimension *D*_max_ was estimated from the *P*(*r*) function as the distance *r* for which *P*(*r*) = 0^35^. The *I*(*q*) and *R*_g_ of 20 models corresponding to the NMR structures were calculated by using the program CRYSOL^37^ and averaged. The molecular weight of the sample was estimated by comparing *I*(0)/*c* (where *c* is the protein concentration) of the sample to that of lysozyme.

### Replica exchange molecular dynamics (REMD) simulations

Replica exchange molecular dynamics (REMD) simulations^38^,^39^ for the four systems, unmod-NCD (amino acids 1–80) and phos-NCD (amino acids 1–80) complexed with the H3K9me peptide (amino acids 1–22), and unmod-NCD and phos-NCD without the peptide, were performed in explicit solvent. The starting structure of the CD region was built by homology modeling using MODELLER^40^ and the crystal structure (PDB: 1Q3L), whereby the structure of residues 60–64 was modified to be an α-helix on the basis of the NMR structures, and the disordered regions (residues 1–20 and 75–80 for NCD, and 1–4 and 17–22 for the H3K9me peptide) were built simply in random coil. The C-termini of both chains were capped by N-methyl group. The protein was immersed in a rectangular box (56.6 × 58.3 × 85.4 Å^3^ on average) filled with TIP3P waters^41^ together with sodium and chloride ions to generate the SAXS experimental ionic concentration, resulting in 40,055, 41,722, 34,683, 36,263 atoms in total for the complex forms of unmod-NCD and phos-NCD, and the apo forms of unmod-NCD and phos-NCD, respectively.

REMD simulations were performed by the MD program MARBLE^42^ with an extension for the REMD (*P* = 1 atms, *T* is described below). The force field used in these computations was the CHARMM 36 all-atom parameter^43^ and the parameters for methylated lysine^44^. Electrostatic interactions were calculated using the particle-mesh Ewald method^45^. The cut-off length of the Lennard-Jones potential was 10 Å. The symplectic integrator for rigid bodies was used to constrain the bond lengths and angles involving hydrogen atoms^42^, allowing the time step to be 2.0 fs. Forty-eight replicas were used in each simulation with temperatures ranging from 290.53 K to 443.15 K with intervals generated in the exponential scale. The highest temperature, 443.15 K, was chosen in order to sample all possible structures including the flexible N-terminal region. To avoid the dissociation of the H3K9me peptide, distance restraints were applied between N_ζ_ of K9me and the centers of mass of the aromatic rings comprising the aromatic cage (residues 20, 41, and 44) with the harmonic force constant of 0.15 kcal/mol/Å^2^. 50-ns simulations were carried out for each of four systems using Metropolis trials to exchange temperatures between neighboring replicas every 10 ps. The simulation trajectories with *T* = 293.15 K (the second lowest temperature for the 48 replicas) were selected from all of the replicas as the structural ensembles for analysis.

## Additional Information

**Accession codes:** Coordinate and structural factors have been deposited in the Protein Data Bank under accession code 2RVL for unmod-NCD, 2RVM for phos-NCD in its unbound state, and 2RVN for the phos-NCD bound to H3K9me. NMR constraints have been deposited in the Biological Magnetic Resonance Bank under entry 11604 for unmod-NCD, 11605 for phos-NCD in its unbound state, and 11606 for the phos-NCD bound to H3K9me. 

**How to cite this article**: Shimojo, H. *et al*. Extended string-like binding of the phosphorylated HP1a N-terminal tail to the lysine 9-methylated histone H3 tail. *Sci. Rep.* **6**, 22527; doi: 10.1038/srep22527 (2016).

## Supplementary Material

Supplementary Information

## Figures and Tables

**Table 1:** Structural statistics table.

	**Phos-NCD**	**Unmod-NCD**	**Complex**
Total distance restraints^a^	739 (74)	652 (53)	637 (46)
Short range (|i–j| ≦ 1)^a^	476 (74)	382 (53)	390 (45)
Medium range (1 < |i–j| < 5)^a^	101 (0)	94 (0)	80 (1)
Long range (|i–j| ≧ 5)^a^	162 (0)	176 (0)	167 (0)
Hydrogen bonds^a^	18 (0)	15 (0)	7 (0)
NOEs between protein and peptide	–	–	43 (20)
Dihedral angle restraints			
Φ^a^	47 (2)	49 (4)	50 (1)
Ψ^a^	47 (2)	49 (4)	49 (1)
Maximum and total constraint violations			
Upper distance limits (Å)	0.0042 ± 0.0007/0.12 ± 0.04	0.0023 ± 0.0005/0.08 ± 0.03	0.0058 ± 0.0010/0.18 ± 0.06
Lower distance limits (Å)	0.0009 ± 0.001	0.0001 ± 0.0002	0.0025 ± 0.0026
van der Waals contacts (Å)	Sum 12.4 ± 0.3/Max 0.25 ± 0.01	Sum 0.7 ± 0.1/Max 0.13 ± 0.01	Sum 15.3 ± 0.4/Max 0.27 ± 0.02
Torsion angle ranges (°)	0.2637 ± 0.0393/1.72 ± 0.40	0.0428 ± 0.0278/0.31 ± 0.28	0.5691 ± 0.0971/3.00 ± 0.61
Average CYANA target function (Å^2^)^b^	2.43 ± 0.051	0.084 ± 0.0137	3.16 ± 0.0722
Average pairwise r.m.s. deviation^c,d,e^			
Backbone atoms (Å)	0.40 ± 0.11	0.42 ± 0.10	0.42 ± 0.09
Heavy atoms (Å)	0.92 ± 0.11	1.02 ± 0.08	0.96 ± 0.08
Ramachandran plot statistics^c,d,e,f^			
Residues in most favored regions (%)	83.9	84.9	85.4
Residues in additional allowed regions (%)	16.1	15.1	14.6

^a^In parentheses, the numbers for N-terminal tail of amino acids 1–19 are shown.

^b^Values for the ensemble of the 20 lowest structures out of 600 calculated.

^c^Phosphorylated residues 21–73.

^d^Un-phosphorylated residues 21–73.

^e^HP1α chromodomain residues 20–73 and histone peptide residues 5–10.

^f^As determined by the program PROCHECK-NMR.
