# Supplementary material for: Extended string-like binding of the phosphorylated HP1α N-terminal tail to the lysine 9-methylated histone H3 tail
Source: Sci Rep. 2016 Mar 3;6:22527. doi: 10.1038/srep22527 (PMC4776139; doi:10.1038/srep22527)
Supplement: Supplementary Information [file srep22527-s1.pdf]

Supplementary Information

Extended string-like binding of the phosphorylated HP1α N-terminal tail to the lysine 9-methylated histone H3 tail

Hideaki Shimojo[1], Ayumi Kawaguchi[1], Takashi Oda[1], Nobuto Hashiguchi[1], Satoshi Omori[1], Kei Moritsugu[1], Akinori Kidera[1], Kyoko Hiragami-Hamada[2], Jun-ichi Nakayama[3], Mamoru Sato[1] and Yoshifumi Nishimura[1]*

[1] Graduate School of Medical Life Science, Yokohama City University, 1-7-29 Suehiro-cho, Tsurumi-ku, Yokohama, Kanagawa 230-0045, Japan

[2] Division of Genome Technologies, RIKEN Center for Life Science Technologies, 1-7-22 Suehiro-cho, Tsurumi-ku, Yokohama, Kanagawa 230-0045, Japan

[3] Graduate School of Natural Sciences, Nagoya City University, 1 Yamanohata, Mizuho, Nagoya, Aichi 467-8501, Japan

*Corresponding author: nisimura@tsurumi.yokohama-cu.ac.jp

a b

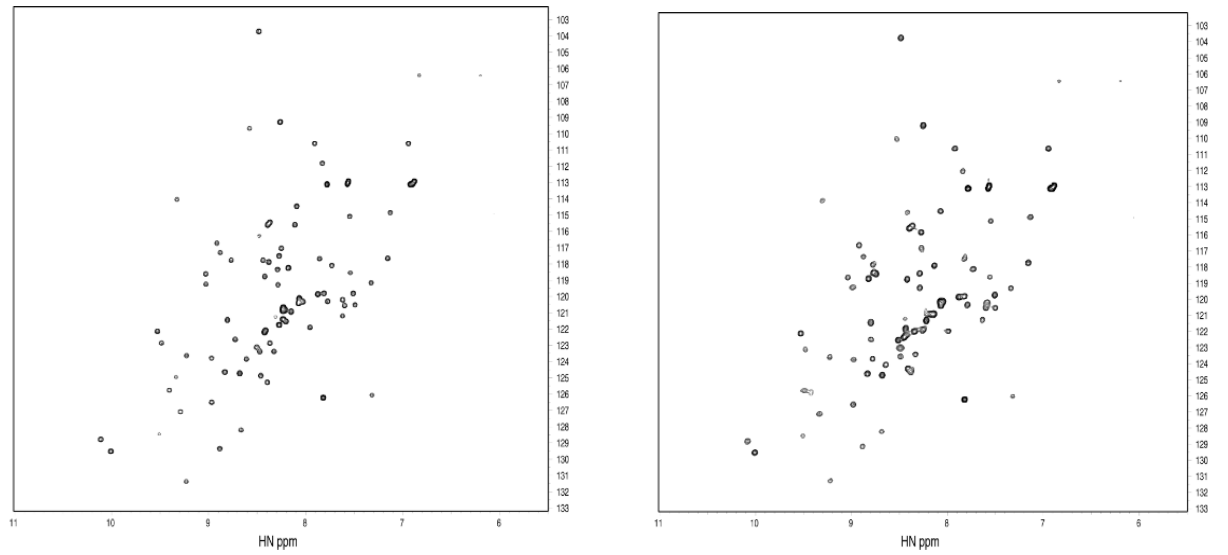

Supplementary Figure S1

HSQC spectra of unmod-NCD (a) and phos-NCD (b).

PROCHECK-NMR

Ramachandran Plot

(20 models)**

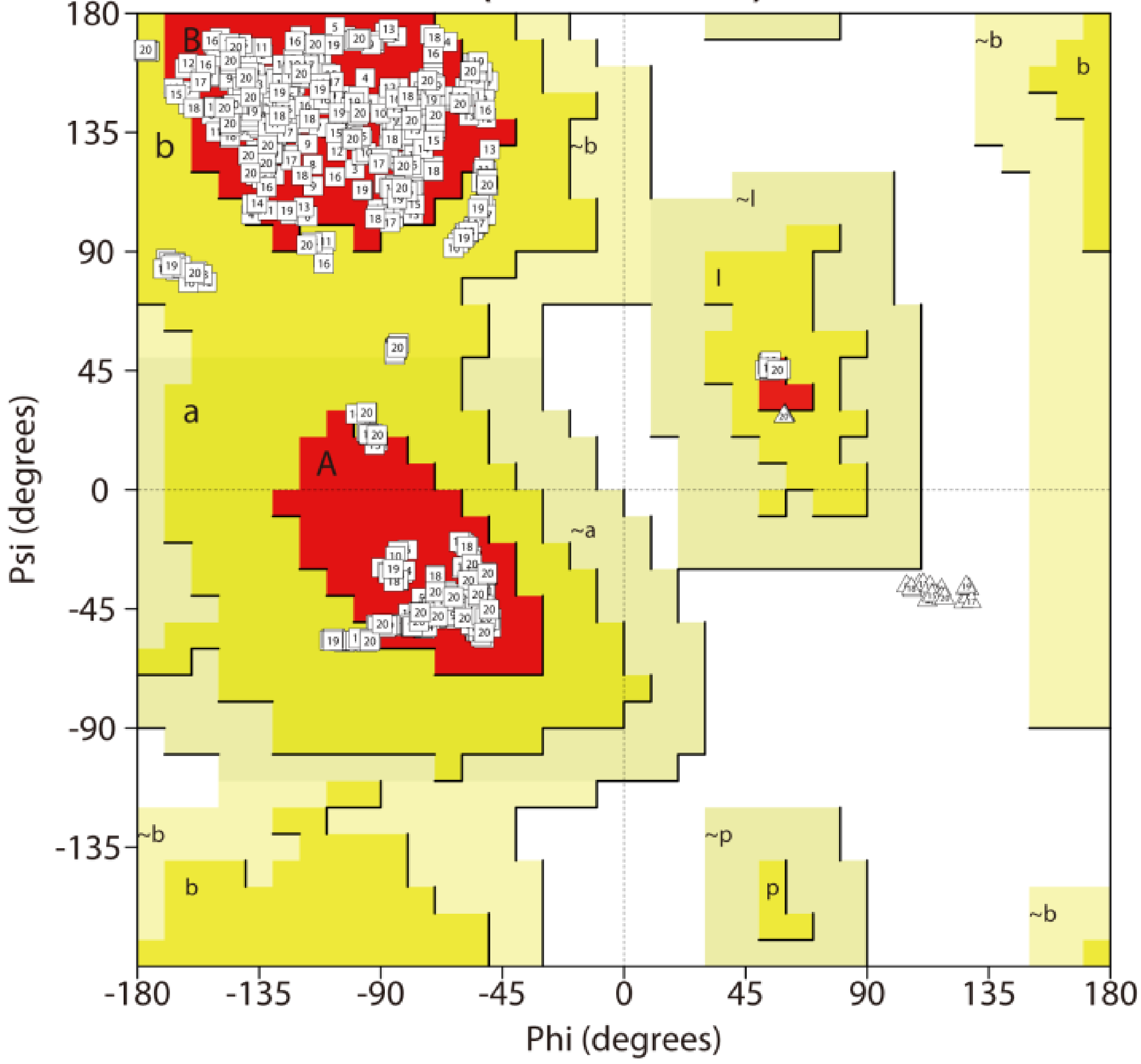

Plot statistics

Residues in most favoured regions [A,B,L]	822	83.9%
Residues in additional allowed regions [a,b,l,p]	158	16.1%
Residues in generously allowed regions [~a,~b,~l,~p]	0	0.0%
Residues in disallowed regions	0	0.0%
	----	------
Number of non-glycine and non-proline residues	980	100.0%
Number of end-residues (excl. Gly and Pro)	0	
Number of glycine residues (shown as triangles)	40	
Number of proline residues	40	

Total number of residues	1060	

Based on an analysis of 118 structures of resolution of at least 2.0 Angstroms and R-factor no greater than 20%, a good quality model would be expected to have over 90% in the most favoured regions.
Model numbers shown inside each data point.

** Selected residues only

Supplementary Figure S2

Ramachandra plot statics is calculated for 20 structures of phos-NCD over residues 21-73 by PROCHCK_NMR.

PROCHECK-NMR

Ramachandran Plot

(20 models)**

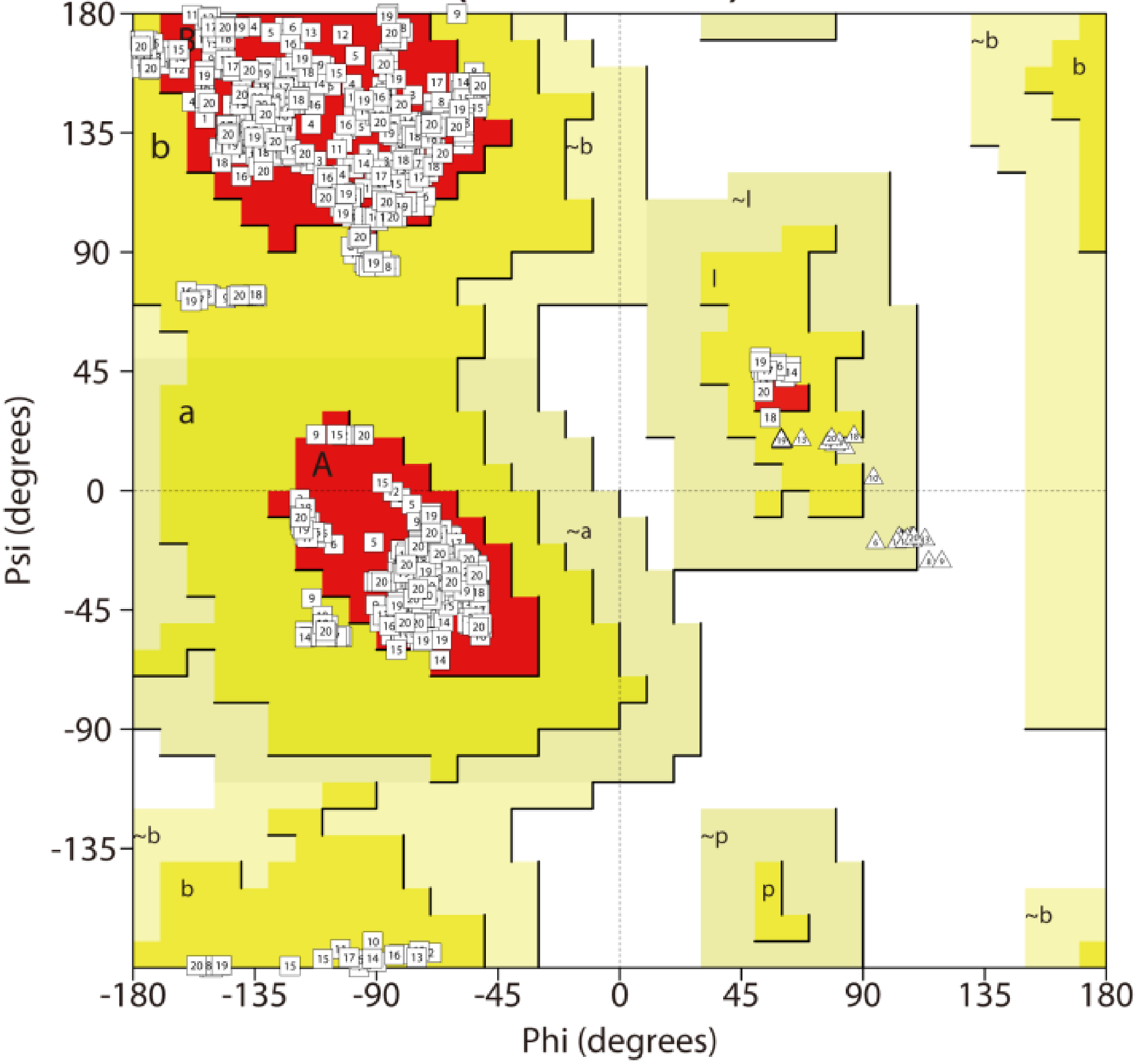

Plot statistics

Residues in most favoured regions [A,B,L]	832	84.9%
Residues in additional allowed regions [a,b,l,p]	148	15.1%
Residues in generously allowed regions [~a,~b,~l,~p]	0	0.0%
Residues in disallowed regions	0	0.0%
	----	------
Number of non-glycine and non-proline residues	980	100.0%
Number of end-residues (excl. Gly and Pro)	0	
Number of glycine residues (shown as triangles)	40	
Number of proline residues	40	

Total number of residues	1060	

Based on an analysis of 118 structures of resolution of at least 2.0 Angstroms and R-factor no greater than 20%, a good quality model would be expected to have over 90% in the most favoured regions.
Model numbers shown inside each data point.

** Selected residues only

Supplementary Figure S3

Ramachandra plot statics is calculated for 20 structures of unmod-NCD over residues 21-73 by PROCHCK_NMR.

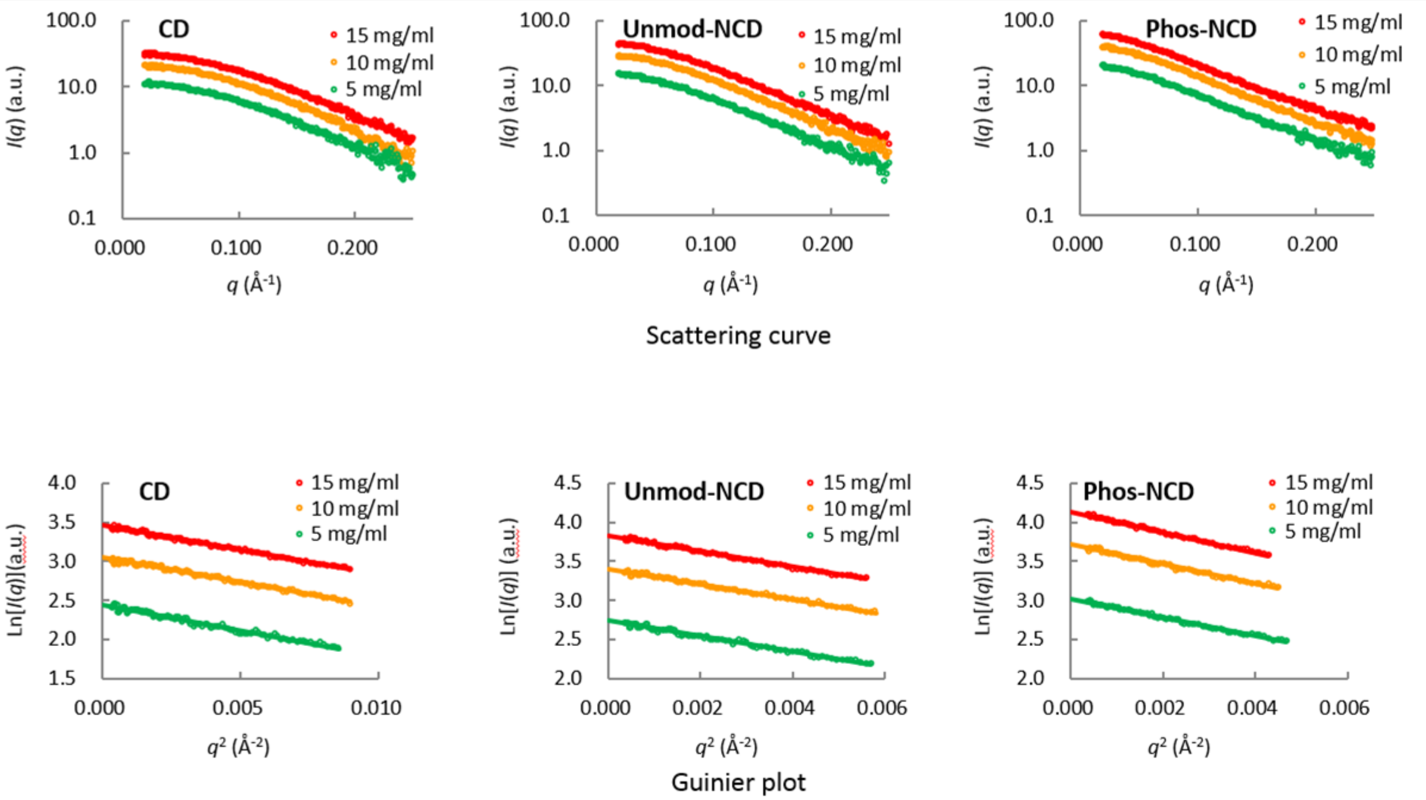

Supplementary Figure S4

Scattering curves and derived Guinier plots of CD, unmod-NCD, and phos-NCD. The SAXS data of scattering curves were collected at three different protein concentrations, 5, 10 and 15 mg/ml.

a

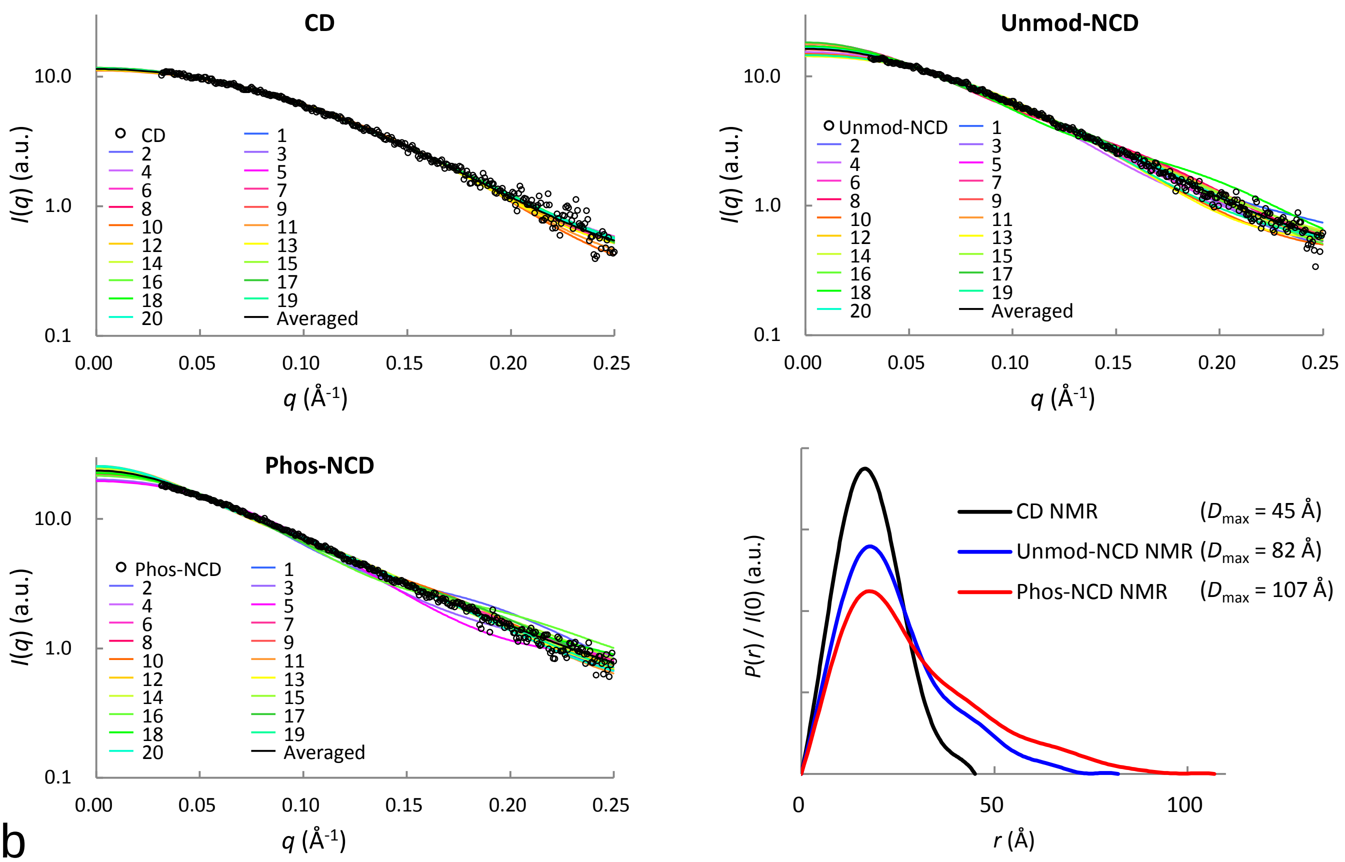

b

	Model No,	1	2	3	4	5	6	7	8	9	10	11	12	13	14	15	16	17	18	19	20	Averaged value
CD	χ^2	0.12	0.10	0.10	0.10	0.11	0.11	0.14	0.11	0.13	0.19	0.15	0.12	0.14	0.10	0.10	0.10	0.10	0.10	0.10	0.10	0.12
	R_g	13.8	14.2	13.9	13.9	13.9	13.8	13.7	13.7	13.6	13.4	13.7	13.8	13.7	14.0	14.1	14.2	14.4	14.1	14.0	14.1	13.9
Unmod-NCD	χ^2	0.36	0.13	0.15	0.42	0.47	0.16	0.56	0.79	0.22	0.30	0.56	0.18	0.44	0.27	0.26	0.20	0.50	0.96	0.29	0.20	0.37
	R_g	20.3	17.0	16.3	16.3	20.2	16.1	19.8	21.8	18.0	15.5	21.7	19.4	15.1	19.5	19.6	20.0	22.9	19.3	15.6	19.7	18.7
Phos-NCD	χ^2	0.59	1.16	0.48	0.46	0.67	0.61	0.32	0.63	0.54	0.58	0.69	0.68	0.49	0.59	0.70	1.01	0.72	0.27	0.38	0.49	0.60
	R_g	26.5	20.5	17.8	26.8	16.8	25.3	20.9	25.6	24.9	21.1	27.2	25.8	26.2	25.0	22.3	19.4	22.2	22.3	27.6	27.9	23.6

Supplementary Figure S5

The calculated scattering curves from NMR structures of CD, unmod-NCD and phos-NCD and derived their P(r) functions (a). CD structures derived from the corresponding part of phos-NCD. (b). Each parameter obtained from the 20 NMR structures.

sample	Kd [μM]	N [sites]	Ka [M^{-1}]	ΔH [cal/mol]	ΔS [cal/mol/K]
unmod-NCD	1.77±0.05	1.09	5.64E5±2.59E4	-6790±45.33	3.16
phos-NCD	0.17±0.02	1.03	5.87E6±5.21E5	-8424±44.0	2.23
NCDΔ1-4	0.76±0.03	1.04	1.31E6±5.07E4	-5933±22.94	7.75
phos-NCDΔ1-4	0.38±0.03	1.03	2.61E6±2.36E5	-8271±59.99	1.14
NCDΔ1-9	0.16±0.02	1.01	6.04E6±2.55E5	-7435±16.79	5.66
phos-NCDΔ1-9	0.04±0.01	1.04	2.58E7±5.24E6	-6233±42.46	12.6
NCDΔ1-14	2.52±0.26	1.00	3.97E5±4.07E4	-7178±114.1	1.13
NCDΔ1-19	13.3±0.41	1.08	7.50E4±2.30E3	-1.036E4±103.0	-13.0

Supplementary Figure S6

Binding affinities of various N-terminal deleted mutants of numod-NCD and phos-NCD by ITC experiments. Derived values for dissociation constant (Kd), stoichiometry (N), enthalpy change (△H) and entropy change (△S) are indicated.

PROCHECK-NMR

Ramachandran Plot

(20 models)**

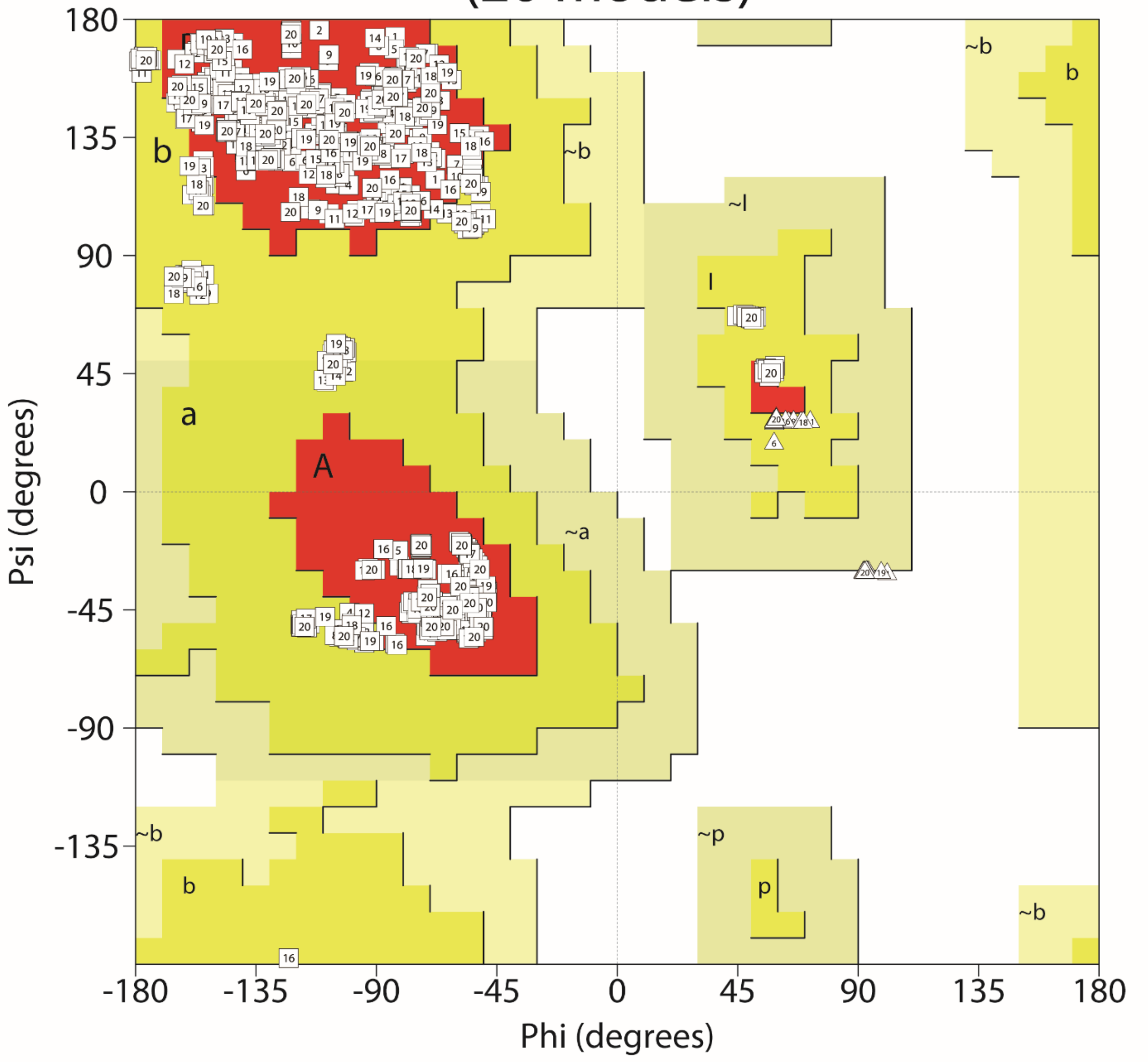

Plot statistics

Residues in most favoured regions [A,B,L]	956	85.4%
Residues in additional allowed regions [a,b,l,p]	164	14.6%
Residues in generously allowed regions [~a,~b,~l,~p]	0	0.0%
Residues in disallowed regions	0	0.0%
	----	------
Number of non-glycine and non-proline residues	1120	100.0%
Number of end-residues (excl. Gly and Pro)	0	
Number of glycine residues (shown as triangles)	40	
Number of proline residues	40	

Total number of residues	1200	

Based on an analysis of 118 structures of resolution of at least 2.0 Angstroms and R-factor no greater than 20%, a good quality model would be expected to have over 90% in the most favoured regions.
Model numbers shown inside each data point.

** Selected residues only

Supplementary Figure S7

Ramachandran plot statistics is calculated for 20 structures of the HP1α-CD/H3K9me3 peptide complex over residues 20-73 of the HP1α chromodomain and residues 5-10 of the histone peptide.

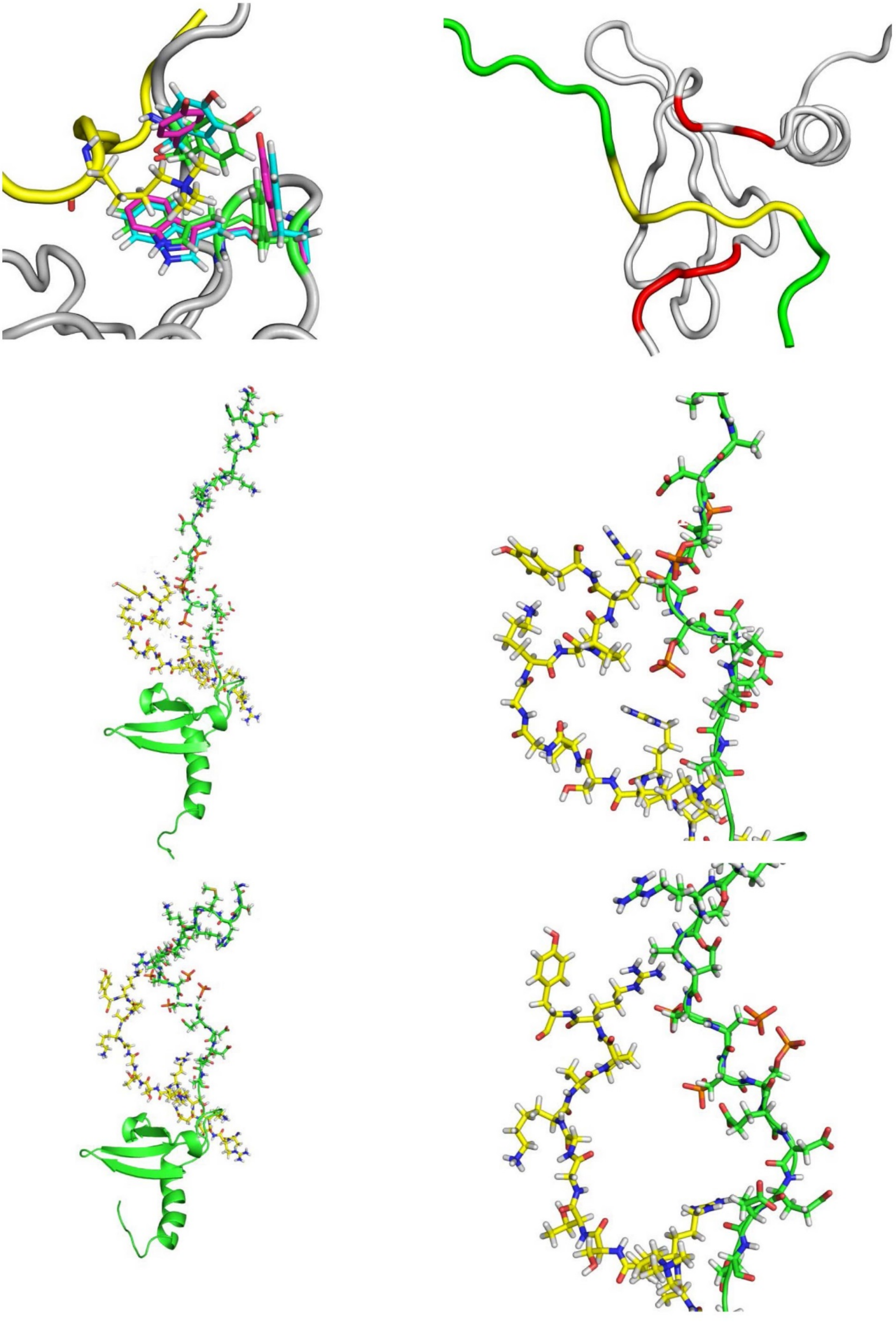

Supplementary Figure S8

Structure of the complex of phos-NCD bound to the H3K9me peptide.

(**a**) Structural comparison of the aromatic cages of H3K9me-bound phos-NCD (green), mouse HP1β (cyan; PDB ID: 1GUW) [12] and *Drosophila* HP1a (magenta; PDB ID: 1KNE) [11]. The backbone of phos-NCD and the H3K9me peptide in the complex are shown in gray and yellow, respectively. (**b**) Interacting amino acids of phos-NCD and the H3K9me peptide. The chromodomain backbone and interacting amino acids of phos-NCD are shown in gray and green, respectively; the backbone and interacting amino acids of the H3K9me peptide are shown in red and yellow, respectively. (**c**) Two typical structures showing plausible interaction modes between the H3K9me peptide and the N-terminal phosphorylated serine and acidic segment of phos-NCD.

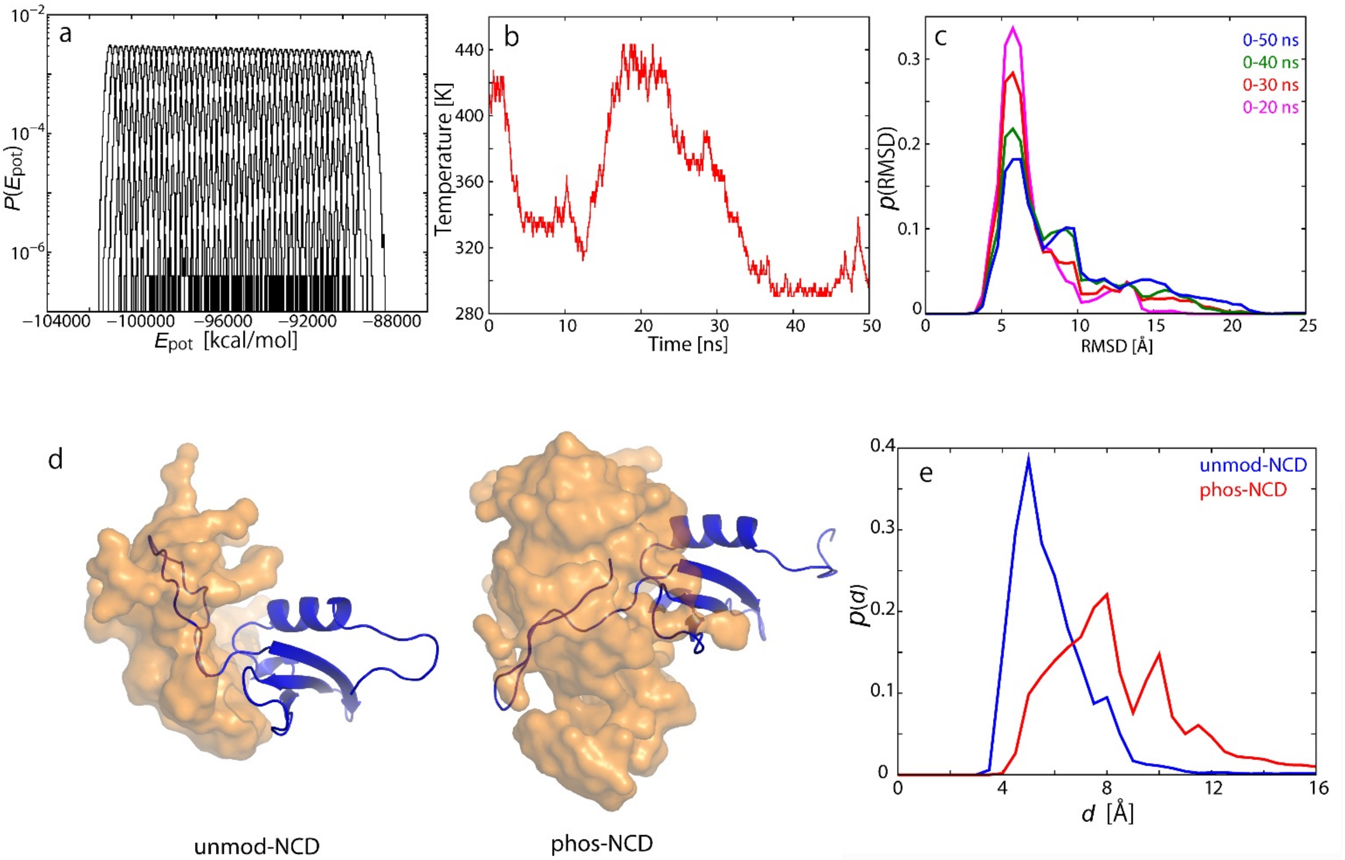

Supplementary Figure S9

(a) Probability distributions of the potential energy V, $P(V)$, for the 48 replicas of the REMD simulation for unmod-NCD with H3K9me. (b) Time course of temperature experienced by a representative replica. (c) The distributions of the Cα RMSD for the N-terminal residues (amino acids 1-20) of unmod-NCD with H3K9me after superimposing the core region (amino acids 20-73), calculated using four different lengths of the trajectory, 0-20 ns (magenta), 0-30 ns (red), 0-40 ns (green), and 0-50 ns (blue). (d) Occupancy maps of Cα atom of Met1 for unmod-NCD and phos-NCD without H3K9me after superimposing the core region (amino acids 20-73), together with snapshots during the REMD simulations. (e) Probability distributions of the minimum distance between Cα atoms of amino acids 1-10 (the edge of N-terminal tail) and those of amino acids 19-21/56-59 (having atom contacts with H3K9me in the bound form), d, for unmod-NCD (blue) and phos-NCD (red) without H3K9me.

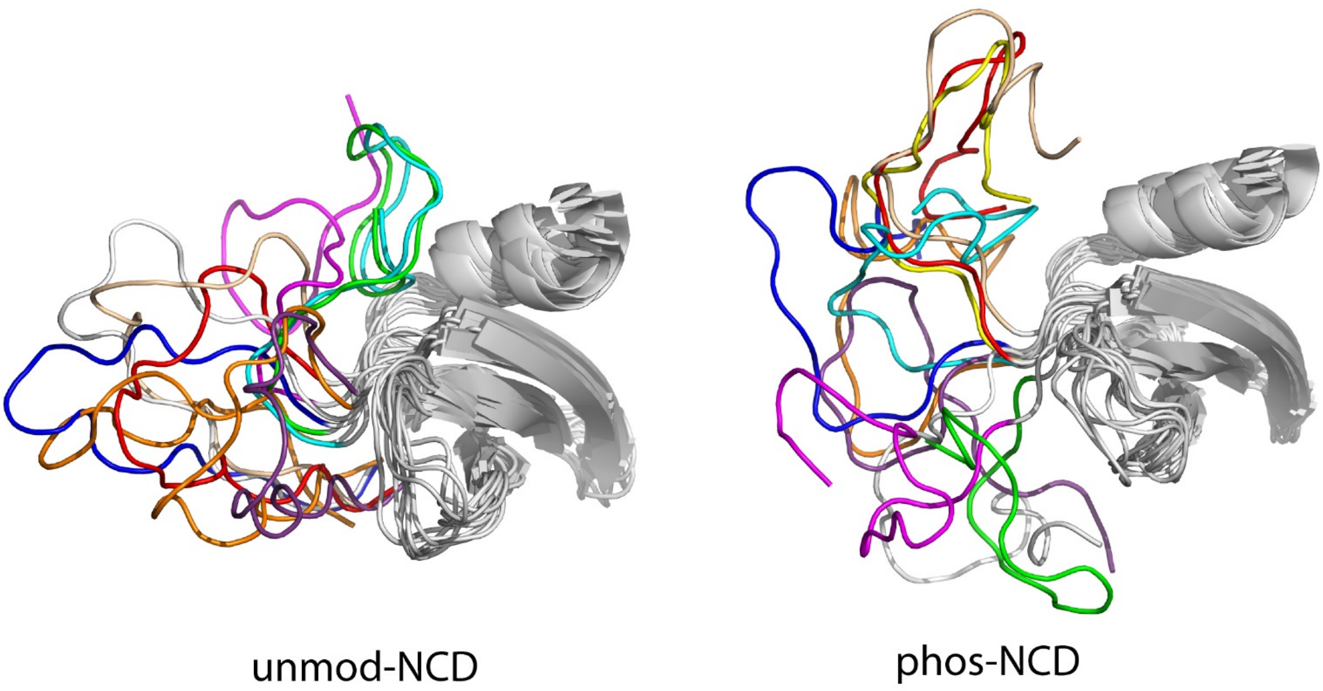

Supplementary Figure S10

Representative structures of unmod-NCD and phos-NCD from REMD simulations.

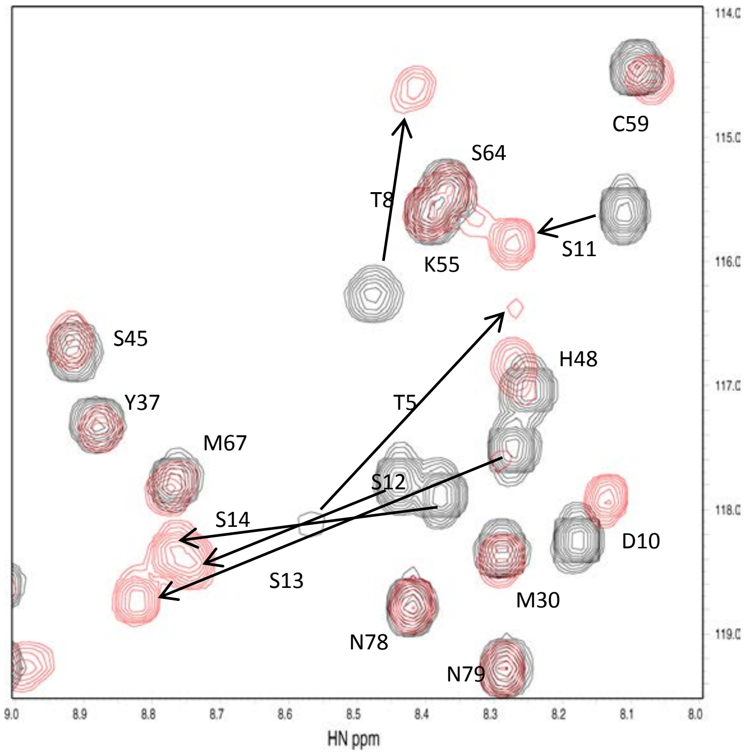

Supplementary Figure S11

HSQC spectra of unmod-NCD (black) and phos-NCD (red).
